# KSHV-Mediated Angiogenesis in Tumor Progression

**DOI:** 10.3390/v8070198

**Published:** 2016-07-20

**Authors:** Pravinkumar Purushothaman, Timsy Uppal, Roni Sarkar, Subhash C. Verma

**Affiliations:** Department of Microbiology and Immunology, University of Nevada, Reno, School of Medicine, 1664 N Virginia Street, MS 320, Reno, NV 89557, USA; pravinp@medicine.nevada.edu (P.P.); tuppal@medicine.nevada.edu (T.U.); rsarkar@medicine.nevada.edu (R.S.)

**Keywords:** Kaposi’s sarcoma-associated herpesvirus, KSHV, Kaposi’s sarcoma, angiogenesis, lymphangiogenesis, oncogenesis, oncoproteins

## Abstract

Human herpesvirus 8 (HHV-8), also known as Kaposi’s sarcoma-associated herpesvirus (KSHV), is a malignant human oncovirus belonging to the gamma herpesvirus family. HHV-8 is closely linked to the pathogenesis of Kaposi’s sarcoma (KS) and two other B-cell lymphoproliferative diseases: primary effusion lymphoma (PEL) and a plasmablastic variant of multicentric Castleman’s disease (MCD). KS is an invasive tumor of endothelial cells most commonly found in untreated HIV-AIDS or immuno-compromised individuals. KS tumors are highly vascularized and have abnormal, excessive neo-angiogenesis, inflammation, and proliferation of infected endothelial cells. KSHV directly induces angiogenesis in an autocrine and paracrine fashion through a complex interplay of various viral and cellular pro-angiogenic and inflammatory factors. KS is believed to originate due to a combination of KSHV’s efficient strategies for evading host immune systems and several pro-angiogenic and pro-inflammatory stimuli. In addition, KSHV infection of endothelial cells produces a wide array of viral oncoproteins with transforming capabilities that regulate multiple host-signaling pathways involved in the activation of angiogenesis. It is likely that the cellular-signaling pathways of angiogenesis and lymph-angiogenesis modulate the rate of tumorigenesis induction by KSHV. This review summarizes the current knowledge on regulating KSHV-mediated angiogenesis by integrating the findings reported thus far on the roles of host and viral genes in oncogenesis, recent developments in cell-culture/animal-model systems, and various anti-angiogenic therapies for treating KSHV-related lymphoproliferative disorders.

## 1. Introduction

Kaposi’s sarcoma-associated herpesvirus (KSHV), also called human herpesvirus 8 (HHV-8), is one of the most recently-discovered human oncogenic viruses and a major cause of aggressive, AIDS-defining malignancies worldwide [1]. KSHV is an enveloped virus containing a large (~165 kb) double-stranded (ds) DNA genome and belongs to the *Rhadinovirus* genus of the *Herpesviridae* family. KSHV is a γ2-lymphotropic-oncogenic virus, classified together with Epstein-Barr virus (EBV), murine gammaherpesvirus-68 (MHV-68), and herpesvirus saimiri (HVS) (reviewed in [2]). KSHV was originally identified from Kaposi’s sarcoma (KS) lesions from an AIDS patient using a representational difference analysis (RDA) technique [3]. Since its discovery in 1994, KSHV has been linked to the development of three neoplastic disorders, primarily KS, primary effusion lymphoma (PEL), or body cavity-based lymphoma (BCBL), and a plasmablastic variant of multicentric Castleman’s disease (MCD) [4,5]. KSHV has also been shown to be associated with several other lymphomas, including germinotropic lymphoproliferative disease (GLD), multiple myeloma, angiosarcomas, malignant skin tumors and squamous cell carcinomas [6]. Recently, a new clinical KSHV-associated syndrome has been identified, KSHV Inflammatory Cytokine Syndrome (KICS), which has clinical manifestations similar to KSHV-MCD [7]. KICS has been proposed to contribute to the inflammatory symptoms seen in patients infected with KS and PEL.

Similar to other herpesviruses, KSHV has a linear, double-stranded DNA genome, which is enclosed within a large icosahedral capsid, enveloped by an amorphous tegument layer consisting of several host and viral proteins and an outer glycoprotein-rich, lipid bilayer (reviewed in [8]). KSHV can infect various cell types [9,10] and exhibit either a lifelong, immunologically silent, latent infection or a transient, productive, lytic infection with distinct viral gene-expression profiles. During latent infection, the KSHV genome is maintained as a circular, extra-chromosomal episome, which replicates along with the host cell in a cell cycle-dependent manner with expression of a few viral genes, including latency-associated nuclear antigen (LANA, ORF73), viral cyclin (vCyclin, ORF72), viral FLIP (vFLIP, ORF71), and microRNAs, whose cooperative effects drive cell survival and proliferation (reviewed in [11]). The latent infection is the predominant infection state of KSHV, and in it the viral genome is maintained at 100–150 copies, which are tethered to the host chromosome. In contrast, during the lytic phase, the virus reactivates from latency leading to the production of infectious virions. Upon reactivation, a full repertoire of lytic viral genes, including ORF50, ORF57, ORF59, K8, ORF40, ORF6, ORF9, viral interleukin-6 (vIL-6, ORFK2), viral G protein-coupled receptor (vGPCR, ORF74), and viral chemokines (vCCL-I/ORFK6 and vCCL-II/ORFK4), are expressed in a temporally-regulated manner [12,13,14]. KSHV-encoded lytic genes are well documented to play a significant role in the secretion of multiple paracrine factors, including cytokines and growth factors, vascular endothelial growth factor (VEGF), interleukin-6 (IL-6), interleukin-8 (IL-8), platelet-derived growth factor (PDGF), fibroblast growth factor 2 (FGF2), and matrix metalloproteinases (MMPs), which induce angiogenesis, lymphatic reprogramming, and inflammatory lesions in uninfected and latently-infected cells. [15]. Both the latent and lytic gene transcription programs of KSHV are proposed to drive tumor progression.

Infection of endothelial cells with KSHV plays an important role in viral dissemination and paracrine induction of angiogenesis in KS lesions. KSHV-infected endothelial cells share the characteristics of transformed endothelial cells, including cell proliferation, chemotactic migration, and invasion [16,17]. Furthermore, KSHV infection can upregulate various cellular signaling pathways to increase endothelial cell proliferation and vascular permeability during angiogenesis and vasculogenesis [18]. Therefore, to control KSHV infection and formulate novel treatment strategies for KSHV-associated diseases, it is very important to elucidate the molecular biology of the cellular and viral factors implicated in KSHV-induced oncogenesis. Inhibitors targeting the mechanisms of KSHV-regulated cancer angiogenesis are thought to be effective therapeutic strategies for treating KSHV-associated malignancies.

## 2. KSHV-Associated Human Malignancies

### 2.1. Kaposi’s Sarcoma (KS)

KSHV is the etiological agent of KS, a highly angiogenic endothelial cell tumor, most commonly seen in sub-Saharan Africa and in immune-deficient patients worldwide [19]. The most common KS tumors have spindle-shaped cells infected with KSHV and are clinically characterized by dark red, brown, or purple patches or plaques found cutaneously, mucosally, or viscerally [20]. These endothelial cells of KS tumors are highly proliferative, and the tumors have increased infiltration of inflammatory cells [21,22]. Several studies have shown that the elongated spindle cells of KS tumors sometimes express vascular endothelial cell markers, including CD31, CD34, and CD36 [23,24]. Recent data suggest that KS spindle cells closely resemble lymphatic endothelial cells (LECs) in that they express LYVE-1, VEGF-R3, and podoplanin markers of the lymphatic endothelium, making it difficult to identify the precursor cell type [25,26]. KSHV has been reported to induce c-kit gene expression in dermal microvascular endothelial (DMVEC) cells, thereby transforming them from a cobblestone-like monolayer to KS spindle cells [27,28].

KSHV is required for the development of KS and nearly all KS lesions harbor KSHV viral DNA in the latent phase, although a portion of infected cells in these lesions undergo lytic reactivation, which is believed to play an essential role in tumorigenesis [29]. The role of KSHV in KS development is complex and involves both latent and lytic genes, many of which are pirated versions of cellular genes (reviewed in [30]). KSHV has been identified in all four histologically indistinguishable, but different, epidemiological variants of KS, including Classical KS, Endemic KS, Iatrogenic/organ-transplant KS, and Epidemic AIDS-related KS (reviewed in [31]). Classic KS (the indolent form) usually presents as lesions in the lower and upper extremities without the involvements of lymph nodes and internal organs and affects elderly individuals of Mediterranean or Ashkenazi origin [32,33]. Endemic KS affects sub-Saharan regions and can be indolent or aggressive. Organ transplant-related KS is a relatively indolent, chronic condition with a rapidly progressing course that involves the lymph nodes, mucosa, and inner organs [34]. AIDS/HIV-related KS is the most frequent and aggressive form, indicating that HIV is a potent co-factor for KSHV tumorigenesis [34,35].

### 2.2. Primary Effusion Lymphoma (PEL)

PEL, or body cavity-based lymphomas (BCBL) is a high-grade, B-cell malignancy, an aggressive form of non-Hodgkin’s B-cell lymphoma closely linked to KSHV infection [36]. PEL is often characterized as a lymphomatous effusion tumor present in various body cavities, including the pleurum, pericardium, and peritoneum [37]. Gene expression analyses of PEL cells has indicated the presence of the KSHV genome with a latent profile [38]. Studies have found all PEL cells to be KSHV-positive and nearly 70%–80% of them are also co-infected with EBV [39,40,41]. Consistent growth of PEL cell lines in culture and easy induction to release infectious KSHV virions have made them a valuable in vitro infection model for understanding cellular and molecular mechanisms of KSHV-induced oncogenesis, although the contribution of KSHV to B-cell malignancy still remains a clinical challenge [40].

### 2.3. Multicentric Castleman’s Disease (MCD)

MCD, also called multicentric angiofollicular hyperplasia, is a rare, polyclonal, remitting-relapsing, B-cell lymphoproliferative disease, characterized by vascular proliferation of the germinal centers of the lymph nodes [42]. KSHV has been associated with the plasmablastic variant of MCD, and these lesions harbor the virus in both latent and lytic forms [43]. KS and MCD may occur together and are most commonly observed in immuno-compromised HIV patients and transplant recipients. MCD can also be found in association with B-cell lymphomas, including PEL and Hodgkin’s lymphomas [44]. However, MCD usually does not co-occur with EBV, unlike PEL, and is driven by deregulated expression of cellular and viral cytokines, interleukin-6 (IL-6), and interleukin-10 (IL-10) [45]. In addition, expression of vFLIP, vGPCR, and Kaposin B can increase the expression of cytokines and VEGF in KS, thereby directly contributing to angioproliferation [43].

## 3. KSHV-Mediated Angiogenesis

Angiogenesis is defined as the process by which new blood vessels are formed from the pre-existing blood vessels in response to numerous mechanical, chemical, and inflammatory stimuli, enhancing tumor survival and progression [46]. Tumor growth and metastasis depend on angiogenesis and lymphangiogenesis. Angiogenesis is an important factor in the progression of cancer, as tumor cells are dependent on neovascularization for oxygen and nutrients to sustain their growth [47]. Angiogenesis is regulated through the balance of pro-angiogenic and anti-angiogenic factors, and these pro-angiogenic factors can be released by a variety of cells, including endothelial cells, monocytes, and tumor cells [48]. During tumor growth, excessive release of angiogenic cytokines and growth factors induces an “angiogenic switch” which stimulates the quiescent, non-proliferating, nearby endothelial cells to grow and promote tumor progression [16].

**Figure 1:**
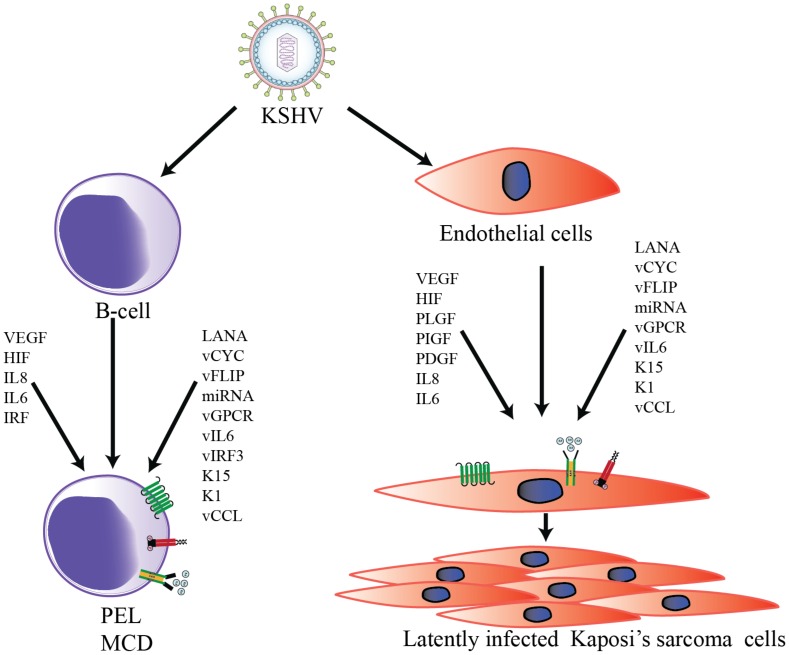
Schematic representation of Kaposi’s sarcoma-associated herpesvirus (KSHV)-induced transformation of B-cells and endothelial cells. KSHV infection activates the expression of multiple viral as well as cellular autocrine and paracrine factors to modulate numerous signaling pathways in order to to promote KSHV-mediated angiogenesis.

Accumulating evidence suggests that KSHV infection can directly induce tumorigenesis through the complex interplay of several viral, cellular angiogenic, and inflammatory markers [49]. KSHV-induced angiogenesis is microscopically visible even during the early stages of KS as leaky, poorly-organized, newly-developed vessels that result in red or purple lesions [50]. Histologically, KS tumors are characterized by abnormal differentiation of endothelial cells into spindle cells, erythrocyte leakage, and vascular spaces, resulting in aberrant vascular structures [50,51]. In cultured endothelial cells, KS infection readily induces angiogenic phenotypes via an elevated secretion of pro-angiogenic factors, including VEGF, IL-6, IL-8, MMPs, Ang2, and Ephrin B2 [52]. In addition, KSHV-infected endothelial cells grown on Matrigel have been shown to form tubules without any external growth factors [52,53]. Furthermore, KSHV-encoded latent and lytic proteins are known to synergistically modulate cellular autocrine and paracrine mechanisms, which contribute to the progression of KSHV-mediated tumorigenesis (Figure 1).

### 3.1. Cellular Factors

Cellular hallmarks of KSHV-mediated angiogenesis, including cellular cytokines, VEGF, and IL-6, are known to readily interact with their corresponding receptors to trigger endothelial cell proliferation [54]. The vIL-6 has been shown to upregulate VEGF expression and angiogenesis in experimental models [55]. Many KSHV latent and lytic proteins play an integral role in inducing angiogenesis and vasculogenesis, [56,57] by activating VEGF and VEGF-R2 [13,58]. It has been hypothesized that upregulation of VEGF during KSHV infection may contribute to a paracrine feedback loop for persistent cellular proliferation and angiogenesis [59,60].

VEGF is an inducer of angiogenesis [61] because it plays a crucial role in vascular permeability, proliferation, and survival of newly-formed vasculature. VEGF-A is a mitogen in endothelial cells in combination with VEGF-R1 and VEGF-R2 [62]. The spindle cells of KS lesions seem to harbor VEGF-A, which is upregulated through inflammatory cytokines in KS lesions [63]. KSHV-positive PEL cell lines produce VEGF-A and, interestingly, it has been reported that capillary morphogenesis in certain endothelial cells can be easily induced if they are treated with conditioned media from these angiogenic cell lines [58,64,65]. In many instances, infection of endothelial cells with KSHV induces angiogenesis, as evidenced by high levels of VEGF-A expression following de novo infection [66,67,68].

In general, hypoxia-inducible factor 1-alpha (HIF-1α) is highly unstable in the presence of oxygen, whereas its stability increases in hypoxic tumors. This allows HIF-1α to trigger the transcription of several genes, including VEGF-A [69]. The function of hypoxia-inducible factors (HIF) is maintained by post-translational modification and stabilization of HIF-1α and hypoxia-inducible factor 1-beta (HIF-1β) proteins [70]. The mRNA of HIF-1α possesses an internal ribosomal entry site that permits translation only under hypoxic conditions [71]. Therefore, KSHV targets HIF-1α for its own advantage, as demonstrated by the augmented expression and stability of HIF-1α in KSHV-infected endothelial cells [72]. The interferon response factor (vIRF-3) encoded by KSHV stabilizes HIF-1α and increases VEGF-A expression [73]. In addition, KSHV manipulates the host glycoproteins initiating the ensuing angiogenic pathways, as observed in the role played by extracellular matrix metalloproteinase inducer (EMMPRIN), a membrane-associated glycoprotein, which increases the expression of VEGF-A during the infection of endothelial cells with KSHV, leading to cellular invasiveness by regulating PI3 kinase and mitogen-activated protein kinases (MAPK) pathways [17,74]. In the context of viral glycoproteins, the expressions of K8.1 and gB have been depicted in latently infected BCBL-1 cells, culminating in VEGF-A expression [65]. Transfection of siRNAs against glycoprotein gB and K8.1 or treatment of these targets with neutralizing antibodies has shown a significant reduction in VEGF-A production.

The sprouting of new blood vessels, remodeling of vasculature, and stimulation of angiogenic factors is governed by many signaling molecules that hold significant juxtapositions within the complex web of signaling pathways. For example, the expression of VEGF receptor is regulated by the PI3K pathway [75]. The heterodimer of PI3K, which comprises a catalytic (p110) and a regulatory subunit (p85), when activated, phosphorylates the effector molecule, AKT, at serine and threonine residues [54,76] stimulating the mammalian target of rapamycin (mTOR) pathway [77,78], which is critical for cell proliferation, gene transcription, protein synthesis, and cell survival [79,80,81], all of which indirectly support angiogenesis. While activating mTOR, AKT adapts various mechanisms, one of which is regulating adenosine triphosphate (ATP) at cellular levels [82]. This inactivates AMPK and Tuberous Sclerosis Complex 2 (TSC-2), and promotes angiogenic pathways. KSHV targets AMPK by suppressing it through the activation of the PI3K/AKT/mTOR pathway, which provides a survival advantage to endothelial cells [68]. In fact, this pathway is critical to the lytic and latent phases of KSHV, and viral proteins have been found to activate this pathway, individually, in both endothelial and B-cells. In addition, activated AKT and mTOR kinases have been identified in both KS and PEL cell lines [83,84,85]. Another pathway that has become significant in the past few decades is the Notch signaling pathway [86]. The downstream effectors of Notch signaling, namely Hey and Hes, have been found to be augmented in KSHV-infected cells [87]. Although earlier studies assumed Notch signaling to be associated with KSHV angiogenesis, its role in tumor growth has now been proven based on the significance of Hey-1 to the development of embryonic vasculatures [88,89]. Through Notch signaling, Hey-1 regulates bone morphogenetic proteins (BMPs), which are active participants in angiogenesis [90,91]. In addition, Hey-1 is highly expressed in KSHV tumor lesions compared to normal tissues [92]. In fact, it has been observed that LANA controls the angiogenic potential of this oncogene by preventing degradation of and stabilizing Hey-1 to cause angiogenesis via the formation of new blood vessels [87].

Among the tumor-suppressor pathways inhibited by KSHV, the Hippo pathway deserves a special mention. This pathway is evolutionarily conserved and comprises a complex network of molecules, primarily LATS1 and 2 kinases [93]. Activated kinases phosphorylate and inhibit Yes-associated protein (YAP) and its closed paralog TAZ (WWTR1) transcription co-activators, which leads to apoptosis and cell suppression [94]. The KSHV virus activates YAP/TAZ (homologous oncoproteins) by inhibiting the Hippo tumor-suppressor pathway kinases, LATS 1 and 2. This inhibition of the Hippo pathway through vGPCR has been shown to be responsible for cell proliferation and tumorigenesis, in a xenograft mouse model [93].

Another interesting cellular proteins are MMPs, a group of enzymes that proteolytically degrades extracellular matrix (ECM) and contribute to angiogenesis by remodeling the ECM during tumor growth, angiogenesis, invasion, and metastasis [95]. KS tumors display an elevated expression of MMP-1, -2, -3, -9, and -19 [96,97], and their possible significance in KS pathology has been indicated by the evaluation of the MMP inhibitor COL-3 in the treatment of AIDS-related KS [98]. The KSHV protein K1 enhances the expression of MMP-9, which in turn directly regulates angiogenesis and tumor progression [60,99]. Analysis of KSHV-infected endothelial cells has indicated an elevated expression of MMP-1, -2, and -9, suggesting their possible role in ECM invasion [52]. Furthermore, it has been shown that KSHV LANA directly activates MMPs by upregulating EMMPRIN [74]. Angiopoietins are another significant family of endothelium-specific angiogenic factors that contribute to KSHV-mediated angiogenesis [100]. The angiopoietin family of VGFs includes, angiopoietin-1, -2, -3, and -4. Angiopoietin-1 (Ang-1/ANGPT-1) directly associates with the Tie-2 receptor tyrosine kinase and upregulates VEGF expression to promote endothelial cell proliferation and blood vessels stabilization. In contrast, angiopoietin-2 (Ang-2) is an antagonist of Tie-2 and destabilizes existing blood vessels [101,102]. Ang-1 is ubiquitously expressed in endothelial cells, whereas Ang-2 has been found to be upregulated at the sites of vascular remodeling [25,100,102,103]. Recently, a study from Keiji Ueda’s group reported that DNA binding factors, including octamer-binding transcription factor (OCT1), play a key role in the upregulation of ANGPT-1 transcriptional activity in PEL cells [104], further indicating that cellular micro environments created by KSHV infection are most probably due to the upregulation of ANGPT-1 expression and may directly contribute to the disease progression in AIDS patients with PEL [104].

Tumor cells often induce angiogenesis by stabilizing HIFs, which are the transcription factors that interact with promoters containing hypoxia response elements (HREs). Interestingly, the key players in angiogenesis, VEGF and VEGF-R1, have been shown to contain HRE [105]. In addition, several viral proteins, including LANA, vIRF3, and vGPCR, can upregulate HIF expression [73,106,107], thereby increasing the levels of HIF angiogenic growth factors and cytokines (VEGF, PDGF, TGFa, TGFb, ANGPT-2, and ANGPTL-4) [106,108]. Additionally, it has been reported that HIF-dependent increases in pyruvate kinase M2 (PKM2) expression, and its upregulation, contribute to angiogenic phenotypes in KS [109]. Activation of both MAPK and p38 kinases that are dependent on KSHV GPCRs leads to subsequent phosphorylation and activation of HIF-1α, which might be considered a plausible mechanism for vGPCR’s induction by VEGF [72,107]. Moreover, KSHV-induced inflammatory processes are likely to play key roles in KS angiogenesis. Elevated expression of cyclooxygenase-2 (COX-2) has been found in KSHV-infected primary endothelial cells, and KS tissues have been reported to play a pivotal role in creating tumor microenvironments during de novo infection [110,111]. KSHV-encoded vFLIP and K15 have been shown to contribute to COX-2-mediated secretion of cellular chemokines and pro-angiogenic factors (IGF1, PDGF, IL14, MCSF, GM-CSF, VEGF-A and -C, angiogenin, oncostatin M, and TGFβ1) [49,111]. Overall, KSHV-induced pro-inflammatory cytokines and angiogenic factors might have evolved to create a tumor microenvironment favorable to viral genome maintenance and oncogenesis [49].

Infection of lymphatic endothelial cells with KSHV has been shown to result in the activation of PI3K/AKT/mTOR signaling pathways mediated through KSHV-encoded lytic proteins, namely, K1, vGPCR, and vIL-6 [112,113,114]. Similarly, KSHV infection in latently-infected PEL cells has demonstrated that PEL cell proliferation and pathogenesis are tightly regulated by a constitutive activation of transcription factor, signal transducer and activator of transcription 6 (STAT6) due to secretion of interleukin-13 (IL-13), downregulation of SH2-containing phosphatase-1 (SHP1), and phosphorylation of Janus kinase inhibitors 1 and 2 (JAK-1/JAK-2) tyrosine kinases [115]. KSHV GPCRs are known to modulate several downstream signaling cascades, including the nuclear factor of activated T-cells (NFAT) pathway. It has been suggested that viral GPCRs promote tumorigenesis by targeting sarcoplasmic reticulum calcium ATPase (SERCA) to elevate cytosolic calcium and induce constitutive activation of the NFAT pathway [116]. Likewise, cellular transforming growth factor-beta 2 (TGF-β2), a cytokine related to TGF-β1, is known to inhibit angiogenesis [117]. In KS tumors and cultured endothelial cells, latent KSHV infection markedly downregulates TGF-β2, but not TGF-β1 mRNA, and induces angiogenic phenotypes, including an enhanced stabilization of capillary-like tube formation [16]. In addition, KSHV infection in cultured cells upregulates enhancer of zeste homolog 2 (EZH2) expression, which is essential for the induction of Ephrin-B2, an essential pro-angiogenic factor that promotes endothelial cell tubule formation [53]. Various other important cellular angiogenic proteins, including IL-1β, FGF-2, HO-1, and PDGF-Rβ, are also highly expressed in KS lesions [118,119].

Recently, for the first time, the functional role of cholesteryl esters (CEs) was demonstrated during the latent and lytic phases of KSHV infection [120]. CEs and triglycerides are common components of lipid droplets found in PEL and non-viral lymphoma cells and seem to be closely linked to the angiogenic properties of the infected cells. These findings suggested that CE metabolism significantly contributes to neo-angiogenesis and reprogramming of KSHV-infected cells and plays a key role in the high metastatic potential of derived tumors [120]. Another recent study has shown that expression of tumor suppressor gene, PDZ and LIM domain protein-2, (PDLIM2) is repressed in KSHV-transformed human umbilical vascular endothelial cells (HUVEC) cells and KSHV-associated cancer cells [121]. In addition, PDLIM2 repression by KSHV is essential for the activation of nuclear factor κB (NF-κB) and signal transducer and activator of transcription 3 (STAT3), for subsequent cellular proliferation and maintenance [121]. Similarly, yet another study has shown that guanine exchange factor switch-associated protein 70 (SWAP70) is crucial for Rac-activation by vGPCR, vGPCR-mediated endothelial tube formation, and endothelial sprouting in vitro [122].

### 3.2. Viral Factors

In infected cells, KSHV can display both the latent and lytic phases of its life cycle [123]. The latent state is considered to be an immune-silent phase, with expression of a limited number of genes needed for episomal maintenance. In contrast, the lytic phase is characterized by the expression of all of the viral proteins. The switch between latent and lytic reactivation is a crucial step in KS pathogenesis [124]. Several KSHV-encoded latent and lytic oncoproteins, including LANA, vCYC, vFLIP, miRNA, K15, KaposinB, K1, K5, vIL-6, vGPCR, vIRF3, vMIPs, and vCCLs, are known to contribute to KSHV-induced aberrant angiogenesis [49]. Table 1 lists some of the important KSHV-encoded proteins and their possible role in KSHV-mediated angiogenesis. 

***LANA*:** LANA, the major latency-associated protein expressed in latently-infected PEL cell lines, has been shown to significantly inhibit p53, the cell cycle checkpoint protein and tumor suppressor [126,174]. LANA also interacts with the G1–S checkpoint proteins, pRB and GSK3β, and modulates G1–S transition [175]. In addition, LANA increases the longevity of primary endothelial cells in culture and makes them less susceptible to apoptosis [127]. LANA has been reported to stabilize and activate the c-Myc oncogene, thereby affecting Myc phosphorylation, stability, transcriptional activity, and apoptotic functions [128,176]. Interaction of LANA with angiogenin (ANG), a multifunctional angiogenic protein, and annexin A2 has been identified in both latently-infected telomerase-immortalized human microvascular endothelium (TIME) and BCBL-1 cells. [177,178]. Upon KSHV infection, LANA has been shown to upregulate the expression of EMMPRIN, a modulator of metastasis and angiogenesis, in primary human fibroblast and endothelial cells [110,179]. Upregulation of EMMPRIN expression induces secretion of IL-6 and VEGF and enhances angiogenesis [17,74]. LANA also stabilizes the Notch effector Hey-1, thereby repressing the expression of Prox-1, a key player in the differentiation of lymphatic endothelial cells [92]. Furthermore, the activation of PDGFRβ, expressed in KS lesions through Notch signaling contributes to the invasive properties of KSHV tumors [51,87,180]. KSHV-encoded LANA also has the potency to inhibit antigen presentation through its acidic central-repeat domain [181,182]. In fact, LANA inhibits immune pathways pertaining to IFN and TNF-α signaling and MHC-I peptide presentation [183,184,185]. In addition, LANA suppresses MHC-II gene expression by interacting with RFX proteins and barring the recruitment of the class II trans-activator CIITA to the site of the MHC-II promoter [186]. Although it is quite apparent that KSHV diminishes antigen presentation, it should still be noted that KSHV also reduces the expression of cell markers, including CD80, CD86, CD1a, and CD83, on the antigen-presenting cells (APCs) [187]. LANA has been reported as being involved in the stability of HIF-1α by (1) inducing the degradation of its suppressors, the von Hippel–Lindau protein and p53 [188]; and (2) interacting with HIF-1α [189].

**v*CYC*:** vCYC, a KSHV-encoded viral homologue of cellular cyclin D [112,190] also contributes to the abnormal characteristics of KS spindle cells [190] and proliferation in PEL cells [137,191]. KSHV-encoded vCYC is expressed together with another latent protein, vFLIP, from a bicistronic mRNA [190,192,193] without the physiological inhibition of cyclins by Cip/Kip or INK4 proteins [190,192,194]. Silencing vCYC or vFLIP by shRNA/siRNA has been shown to induce apoptosis in PEL cells [137]. vCYC, together with the cellular cyclin-dependent kinase CDK6, mediates the phosphorylation of CDC6 and Rb, increases DNA synthesis and triggers progression toward the S phase of the cell cycle [195,196].

**v*FLIP*:** Herpesviral FLICE Inhibitory Protein (vFLIP), encoded by KSHV ORF K13, is structurally related to the death effector domain (DED) and protects against apoptosis induced by Fas/CD95 and TNF receptors [197,198,199]. It has been reported that KSHV-encoded vFLIP induces NF-κB signaling and suppresses Fas-induced apoptosis, suggesting that vFLIP functions primarily by activating classical and alternative NF-κB pathways [200,201,202]. Thus, these studies clearly demonstrate that vFLIP plays an important role in maintaining long-term latency and has the potential to induce pro-inflammatory and angiogenic cytokines, including IL-6 and IL-8.

***miRNA*:** KSHV encodes 12 microRNAs, the majority of which are located between the latently-expressed ORFs 71 and K12 gene in the genome. In addition, many of them have been shown to contribute to KSHV-mediated angiogenesis [203,204,205,206]. All 12 KSHV miRNAs are oriented “in sense” with ORFs 71 and K12 and are expressed primarily during latency [207], although, some have been detected during lytic infection as well [139,207,208,209]. KSHV-encoded miRNAs play a significant role in growth, signaling, and angiogenesis [210,211,212]. It has been shown that the KSHV-encoded miRNA miR-K12-3 directly activates G Protein-coupled receptor kinase 2 (GRK2) to upregulate the migration and invasion of endothelial cells by activating the CXCR2/AKT signaling axis [140]. Similarly, expression of KSHV-encoded miR-K10a, alone, has been found to be sufficient to transform cells, probably through repressing miR-142-3p targets, which have been shown to inhibit transformation [213]. Downregulation of TGFβ signaling plays a significant role in promoting cell proliferation during KSHV infection. KSHV-encoded miR-K12-11 targets SMAD5 to downregulate TGFβ signaling, promoting cell survival and progression [141]. In addition, inhibition of miR-K12-11 has been found to de-repress TGFβ signaling in KSHV-infected B cells [141]. TGFβ signaling has been regulated by thrombospondin 1 (THBS1), a target of the KSHV-encoded miRNAs miR-K12-1, miR-K12-3, miR-K12-6, and miR-K12-11 [142]. THBS1 is an anti-angiogenic factor, and its downregulation leads to repression of TGFβ signaling [205]. Furthermore, miR-K12-6 and miR-K12-11 target the cellular transcription factor MAF to reprogram the blood vessel endothelial cells (BECs) and LECs [143]. Downregulation of MAF by the miRNAs increases the expression of BEC marker genes in the KS tissues. KSHV-encoded miRNAs also repress the expression of breakpoint cluster region protein to enhance Rac1 activity and promote in vitro angiogenesis [214]. Thus, KSHV-encoded miRNAs are capable of altering growth signaling pathways and increasing angiogenesis in support of KSHV-associated tumors [214,215].

***Kaposin B*:** The KSHV protein Kaposin B, translated from the DR repeats and K12 interacts with MK2 kinase via the DR2-encoded sequences, thereby enhancing its activity [151]. Kaposin B interacts with the “C-lobe” region of MK2, a region also targeted by p38 kinase [151]. MK2 activation leads to stabilizing high-turnover cytokine mRNA, including pro-inflammatory and angiogenic IL-6 [151]. In addition, Kaposin B stabilizes the PROX1 mRNA, the “master regulator” of lymphatic endothelial cell differentiation [152]. Stabilizing PROX1 mRNA defines a mechanism by which KSHV infection reprograms the blood-to-lymphatic endothelial marker transition, which is believed to be a critical process in KS development [152,216,217].

***K15*:** K15, another KSHV ORF consists of eight exons located at the right end of the KSHV genome, between ORF75 and the terminal repeat (TR) region. K15 is predominantly expressed during the lytic cycle, but some K15 transcripts have been detected in resting PEL cultures [218,219,220]. Cellular signaling pathways activated by K15 include the Ras/MAPK, JNK/SAPK, and NF-κB pathways and the NFAT/AP1 transcription factors [221,222,223], and induce an angiogenic and pro-inflammatory response. This signaling induces the transcription of a number of cellular cytokines and chemokines, including IL-6, IL-8, CCL20, CCL2, CXCL3, IL-1a/b, and COX-2 [147,224]. Depletion of K15 from the KSHV genome severely affects virus-induced angiogenesis in primary endothelial cells [144]. Kaposi’s sarcoma-associated K15 protein, via its SH2-binding motif, also regulates expression of miR-21 and miR-31 to promote cell migration and invasion [145,220]. Furthermore, in KSHV-infected primary endothelial cells, K15 binds to PLCγ1 and activates calcineurin and NFAT1 to upregulate the expression of host factor RCAN1/DSCR1, inducing angiogenesis and endothelial tubule formation in Matrigel-based assays [144]. Thus, the pro-survival and paracrine-mediated, pro-angiogenic roles of K15 may contribute to KSHV-induced tumorigenesis.

**Figure 2:**
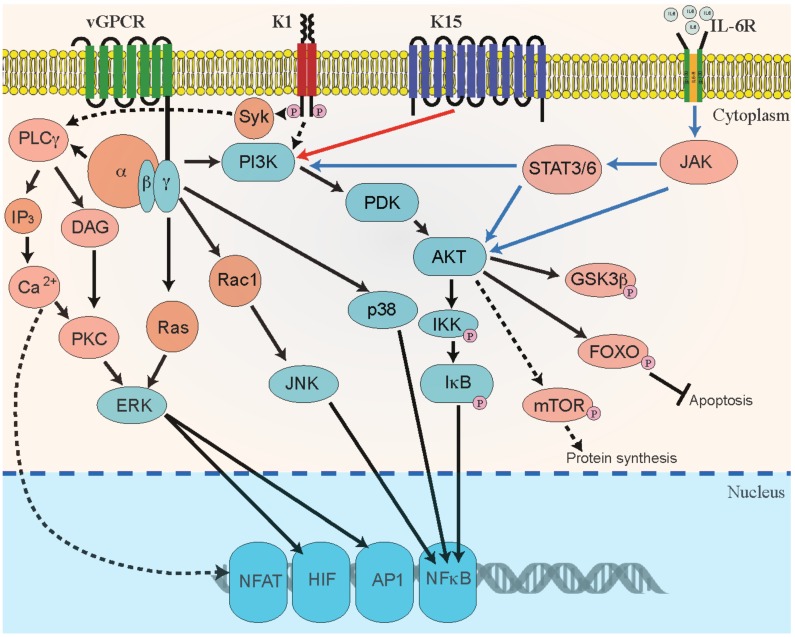
Schematic representation of KSHV-mediated activation of angiogenic signaling pathways. Signaling pathways that are regulated by KSHV proteins viral G protein-coupled receptor (vGPCR), K1, K15, and vIL-6 in B-cells and endothelial cells contribute to KSHV-mediated cellular transformation and angiogenesis through autocrine and paracrine mechanisms. KSHV GPCR and K1 promote cellular signaling through phosphatidylinositol 3-kinase (PI3K), mitogen-activated protein kinases (MAPK) phosphoinositide-dependent kinase (PDK), AKT/protein kinase B (AKT/PKB), and mTOR signaling pathways. Activation of these signaling pathways stimulates the activity of various cellular transcription factors, such as activating protein-1 (AP-1), nuclear factor (NF)-B, hypoxia-inducible factor-1 (HIF1) and Nuclear factor of activated T-cells (NFAT). These transcription factors, in turn, upregulate the secretion of pro-angiogenic growth factors. Signaling through KSHV GPCR is shown as solid black lines and signaling through K1 is shown as black dotted lines. The cellular signaling mediated through K15 and IL-6 receptors are shown as solid red and blue lines, respectively.

***K1:*** K1, a variable ITAM-containing protein (VIP) is a transmembrane glycoprotein, encoded by the first ORF of KSHV. K1 increases the angiogenic characteristics in cultured primary endothelial cells [68,153] and induces angiogenesis by upregulating VEGF production in primary human endothelial cells [153,225,226,227]. K1 signaling can activate secretion of inflammatory cytokines, including IL-6, GM-CSF, IL-1b, IL-8, and IL-10, which are directly implicated in development of KS lesions [225,228]. K1 binds to the m-chain of B-cell receptors (BCRs) to retain the complex in ER and decrease the surface expression of BCRs, thereby improving the longevity of B-cells [229]. Additionally, K1 activation of AKT results in inhibiting the pro-apoptotic forkhead (FKHR/FOXO) transcription factor family, which protects the cells from FKHR- and Fas-mediated apoptosis [113]. Similarly, miRNA-891a-5p mediates synergistic induction of angiogenesis by HIV-1 Tat and KSHV K1 through NF-κB signaling [230]. It has been shown that K1 recruits and activates the Src-family kinases PI3K and PLCγ to mediate signal transduction via several pathways, including ligand-independent, constitutive signaling [231,232]. In KS, PEL, and MCD, K1 exerts a paracrine influence on latently infected and uninfected neighboring cells [233,234]. Overall, these studies suggest that K1 is a multifunctional protein that can constitutively activate multiple pro-growth signaling pathways in KSHV-infected cells. In addition, KSHV acts to promote angiogenesis when co-infected with HIV. The KSHV K1 protein has been reported to act synergistically with the HIV-1 regulatory protein, NEF to induce cell proliferation, vascular tube formation and excessive angiogenesis, in a chicken CAM model [235]. The regulation of angiogenic properties is accomplished by activating PI3K/AKT/mTOR signaling and by downregulating phosphatase and tensin homolog (PTEN) [235]. Since PTEN dephosphorylates PIP3 to PIP2 and inhibits AKT signaling (in other words, cell proliferation), KSHV chooses this molecule to promote tumor formation. PTEN suppression is mediated through the combined effect of HIV-1 Nef and KSHV K1 proteins, which induce cellular miR-718, thereby targeting the sequence in the 3′ UTR of the PTEN’s mRNA, leading to its inhibition [235]. In a nutshell, KSHV endorses cell proliferative pathways through different signaling molecules for its own interest, which adds up to viral angiogenesis (Figure 2).

***K5*:** KSHV-encoded K5, also called modulator of immune recognition (MIR-2), is a viral E3 ligase that is capable of ubiquitinating MHC-I cytoplasmic tail to trigger the internalization and proteasomal degradation of MHC-I complex [236,237,238]. Overexpression of K5 in human dermal microvascular endothelial cells (HDMEC) has been shown to downregulate ICAM-1 expression and block T-cell recruitment or capture [157,239,240]. K5 also degrades VE-cadherin and disrupts VE-cadherin/β-catenin signaling and promotes remodeling of endothelial adherens junctions to initiate angiogenesis [159,241]. K5 protein can activate IFNγ receptor 1, thereby leading to the receptor’s degradation. In addition, K5 suppresses IFNγ-mediated activation of the JAK/STAT pathway [242]. K5’s ubiquitin ligase activity leads to enhanced aerobic glycolysis, thereby initiating lactase production. This K5 activity is mediated through the endocytosis of cellular growth factor-binding receptor tyrosine kinase, which leads to modulation of AKT and extracellular signal-regulated kinase 1 and 2 (Erk 1/2) phosphorylation [243].

***vIL-6*:** KSHV viral interleukin-6 (vIL-6) encoded by ORF K2 shares 24.8% of its amino-acid sequence identity (49.7% similarity) with its cellular counterpart, human IL-6 (hIL-6) [160,244,245]. Nude mice injected with cells stably expressing vIL-6 reportedly grew highly-vascularized tumors [55]. HIV-1-encoded Nef protein [246] can also synergistically enhance vIL-6-mediated angiogenesis. Furthermore, vIL-6 seems to have a dual anti-apoptotic and proliferative effect, as depletion of vIL-6 through shRNA knockdown has reduced the growth rate in KSHV-infected B-cell lines [223]. Similar to cellular IL-6 (cIL-6), vIL-6 could induce gp130 and several other downstream signaling pathways, including JAK/STAT, MAPK, and PI3K/AKT pathways [161,247]. These pathways modulate multiple transcription factors and response elements (REs), including STAT1/3 and STAT5 IL-6 RE, C/EBP, and c-jun promoter IL-6 RE (JRE-IL-6) [248]. Contrary to its defined autocrine role in PEL pathogenesis, vIL-6 is believed to generate KS and MCD primarily by paracrine signaling. Following the induction of RTA expression, vIL-6 is rapidly produced in de novo KSHV-infected cells and in cells undergoing lytic reactivation [249]. In KS lesions, large populations of KSHV-infected cells maintain a latent phase, however, a very small population of cells remain lytically-active. These cells express lytic proteins, including vIL-6, vGPCR, and K1, and ultimately upregulate the expression of cellular inflammatory and angiogenic cytokines [250], including VEGF [251], IL-6, CXCL8 and bFGF, which play important roles in KS development in a paracrine fashion. These secreted effector molecules determine the survival, proliferation, and angiogenesis of KSHV-mediated oncogenesis [252].

***vGPCR*:** KSHV-encoded G-protein-coupled receptor (vGPCR) [253,254] is a viral homologue of the cellular angiogenic IL-8 receptor [166,255]. It is an early lytic protein that significantly contributes to PEL, MCD, and KS development in a paracrine fashion [256,257]. vGPCR modulates cellular signaling through a variety of pathways, including PLC, PKC, MAPK, PI3K/AKT/mTOR, NF-κB, AP1, and NFAT networks, thereby regulating the secretion of many angiogenic factors, primarily, VEGF, bFGF, IL-1β, IL-2, -4, -6, and -8, and TNFα [110,257,258]. In turn, these modulators act in a paracrine fashion and can alter the extracellular microenvironment toward KS tumor progression [54,258]. Endothelial cell-specific expression of vGPCR in Tie2-TVA transgenic mice has led to the formation of multifocal and aberrantly-vascularized tumors with histological similarities to KS [112,258]. These results suggest that vGPCR induces the transformation of cells by modulating the paracrine secretion of pro-inflammatory cytokines and angiogenic growth factors [112,258]. vGPCR has the capability to induce HIF-1α activity through the MAPK and p38 signaling pathways, leading to the phosphorylation of HIF-1α [119]. Signaling through vGPCR causes cellular survival and stimulation of pro-angiogenic signaling pathways [13,83,119,254,259]. KSHV manipulates vGPCR for its own survival benefit and tumorigenesis.

***vIRF-3*:** KSHV-encoded viral interferon regulatory factor (vIRF-3) is a cellular interferon regulatory factor homolog that regulates cellular IRFs and inhibits innate responses from the cell [260]. Unlike other vIRFs, vIRF-3 is consistently expressed as a latent protein in latently-infected PEL cells and has been referred to as latency-associated nuclear antigen-2 (LANA2) [168,261,262]. In PEL cells, vIRF-3 plays a significant role in maintaining latency and pathogenesis [170,171]. HIF-1α, a major regulator of VEGF-A [73,263], is controlled by vIRF-3 by direct interaction, which leads to the stabilization of HIF-1α and aids its nuclear accumulation [73]. In addition, vIRF-3 encourages VEGF production, which promotes angiogenesis. The induction of VEGF through vIRF-3 could be mediated through HIF-1α. The involvement of vIRF-3 in promoting endothelial tube formation in HUVEC cells has been attributed to the production of VEGF [73]. Importantly, vIRF-3 has also been found to activate c-Myc-directed transcription and to decrease the expression and stability of the tumor suppressor protein p53 [128,264,265]. The pro-survival roles of vIRF-3 may also be due to the inhibition of PML-mediated repression of survivin [266]. Altogether, vIRF-3 activities are likely to be critical to maintaining latency and defining PEL malignancy [169,267].

***vCCLs*:** KSHV ORF K6, ORF K4, and ORF K4.1 encode for three homologues of cellular chemokines; viral CC-chemokine ligand-1 (vCCL1/vMIP1), ligand-2 (vCCL2/vMIP2), and ligand-3 (vCCL3/vMIP3), respectively [57,172]. KSHV-encoded v-cyclin, a homolog of cellular cyclin D2, activates cellular CDK6 and promotes G1/S phase transition of the cell cycle. Virus-encoded v-cyclin has been reported to have oncogenic potential, as it induces DNA damage, apoptosis, and autophagy [268]. KSHV-encoded v-cyclin has been shown to interfere with normal T-cell development and to induce lymphoma through v-cyclin-CDK6 complex and Notch activation in vivo [268]. The role of v-cyclin in preventing the cellular senescence and G1 phase arrest induced by HTLV-1 Tax and vFLIP has also been reported [269]. The nature of the viral-chemokine-targeted receptors indicates that they may mediate immune evasion by polarizing Th2 and blocking leukocyte trafficking, as demonstrated for vCCL-2 in in vivo experiments [57,173]. Apart from these immune evasion properties, v-chemokines have also been shown to promote angiogenesis by inducting VEGF [57].

## 4. Mouse Models for Studying KS-Angiogenesis

Mouse models are considered primary in vivo tools used in biomedical research to identify molecular targets and pathways implicated in neo-angiogenesis and tumor progression. In addition, mouse models help validate the efficacy and safety of anti-angiogenic therapies before they are tested in clinical trials. Tumor-transplanted xenograft mouse models have been immensely valuable in understanding the role of angiogenesis and various angiogenic factors during several stages of tumor development [270]. Although significant progress has been made in characterizing KSHV tumor progression, lack of a good small animal model for KS pathogenesis has hampered deeper understanding of specific mechanisms of KSHV contribution to the oncogenic process. At present, the major problem faced by KSHV in vivo mouse models is that murine cells do not support the complete viral replication and infection program [271].

KSHV-encoded vGPCR, a homologue of the IL-8 receptor, plays an indispensable role in KSHV-mediated angiogenesis and tumor development. As an interesting approach to studying KS tumor development and pathogenesis in mice, Zhang et al. have recently developed a recombinant murine gamma herpesvirus (γHV68) carrying KSHV vGPCR [272]. Mice infected with this recombinant γHV68 developed angiogenic, inflammatory features that closely resembled human KS. Mice infected with recombinant γHV68 carrying vGPCR could potentially serve as an important model for studying angiogenesis and tumorigenesis induced by human gamma herpesvirus in the context of viral infection. Another group has explored the functional role of the KSHV ORFK1 gene on lymphoproliferation and Fas-mediated apoptosis in transgenic mice [273]. Histological evaluation of K1 transgenic mice indicated the development of lymphoid hyperplasia and splenomegaly, as observed in lymphoma, MCD, and angiosarcoma, suggesting that K1 may contribute to the development of KSHV-associated cancers [273].

In addition, several other mouse models have been developed recently (reviewed in [274]) to study KSHV infection and replication. A major advancement in KSHV research was the generation of the humanized-bone marrow, liver, and thymus (hu-BLT) mouse model to test KSHV infection with recombinant KSHV (rKSHV.219) via various natural routes of infection, including the oral mucosa and intra-vaginal routes [271]. These results showed that KSHV could establish robust latent and lytic infections in human B-cells and macrophages. Therefore, humanized mice may become a promising model for studying KS infection in vivo and routes and extents of viral infection in infected hosts [271]. Similarly, another research group has recently developed two new murine models for studying KS infection and pathogenesis. Murine bone marrow-derived endothelial cells were transfected with bacterial artificial chromosome BAC36 (mECK36 cells) to create a stable population of mECK36 cells, which were subsequently injected into immuno-deficient mice [275]. Even though mECK36 sarcomas consisted of latently- and lytically-infected spindle cells, for some reason these mice were not able to produce infectious virions. When the BAC36 in the mECK36 cells were replaced with rKSHV.219 and injected into the mice, they developed tumors that produced herpesvirus-like particles, as observed by electron microscope [275]. In addition, to evaluate the ability of these cells to support lytic replication, they were treated with trichostatin A (TSA), a histone deactetylase (HDAC) inhibitor, to induce viral reactivation. These models expressed several KSHV lytic genes and productively infected tumors in vivo, suggesting that they could be used therapeutically to test targeted antiviral compounds.

## 5. Current Treatment Strategies for KS Tumors

The growth of functional vessels during angiogenesis requires a synergistic interaction between numerous endothelial growth factors, receptors, and multiple cellular-signaling pathways [276]. A better understanding of this process enables identification of potential targets for inhibiting neovascularization [277]. In the case of KSHV-induced angiogenesis and oncogenesis, targeting either the cellular and viral proteins with a postulated link to angiogenesis or the virus-mediated signaling pathways, may provide a new treatment for KSHV-mediated, aberrant angiogenesis of endothelial cells. Interestingly, a promising new study has shown that Fumagillin, a potent natural angiogenesis inhibitor, induces KSHV lytic/RTA gene expression and KSHV genome replication and inhibits cell growth in stimulated PEL cells [278]. The inhibitory behavior of TNP-470, a synthetic analog of Fumagillin, on angiogenesis has been predicted to be associated with the upregulation of p21 expression in endothelial cells by activating p53 pathways [279]. Current options for tumor therapies include chemotherapy, cytotoxic drugs, combined anti-retroviral therapy, and immune modulators. A general approach to targeting tumor angiogenesis involves using either anti-VEGF monoclonal antibodies or tyrosine kinase inhibitors (TKIs) [277]. Most of the compounds with anti-angiogenic activities that enter the drug-development process have been reported as targeting the VEGF ligand or its receptors/VEGFRs. Several multi-targeted TKIs that block the signaling of pathways, including VEGFs, PDGFs, and c-kits, have been developed and approved for treatment of other malignant tumors [45]. Hence, it is appropriate to use these approved anti-angiogenic drugs to treat KS. Table 2 lists some of the commercially available anti-angiogenic drugs currently used to treat KSHV-mediated cancers.

## 6. Summary

The growth of new blood and lymphatic vessels is important to the progression and metastatic spread of cancer, and this growth occurs through two significant processes: angiogenesis and lymphangiogenesis. Angiogenesis is mediated by several cellular-signaling pathways, with differential expression of pro-inflammatory and pro-angiogenic chemokine factors reflecting the growth and spread of cancerous cells. The highly vascular nature and extensive neovascularization of KS tumors indicate that KSHV directly induces angiogenesis in KS lesions in a paracrine fashion. KSHV-mediated angiogenesis plays an important role in the control of KS tumorigenesis, and its inhibition with anti-angiogenic drugs/agents is considered a valuable therapeutic approach. For several years, research efforts have focused on investigating various cellular-signaling pathways, particularly growth factor-associated angiogenic signaling, contributing to KS tumor growth, proliferation, and invasion. Although a link between KSHV infection and angiogenesis has been suggested, it is still unclear which factors actually drive angiogenesis during KS progression. A thorough investigation of these mechanisms could lead to targeting specific signaling pathways that are directly involved in regulating KSHV-mediated angiogenesis. This, in turn, may help to revolutionize current therapeutic approaches and aid in designing novel, targeted, anti-angiogenic strategies to treat KS.

## Figures and Tables

**Table 1:** Kaposi’s sarcoma-associated herpesvirus (KSHV)-encoded proteins and their role in KSHV-mediated angiogenesis.

KSHV Gene	KSHV Protein	Function	Reference
LANA	Latency Associated Nuclear Antigen	Apart from KSHV genome persistence, it inhibits p53, pRB and extends the lifespan of latently infected cells	[125,126,127,128,129]
vCYC	Homologue of cellular cyclin D	Primarily regulates cell cycle and promotes oncogene-induced senescence	[130,131,132,133,134]
vFLIP	Homologue of FLICE inhibitory protein	Regulates activation of NF-κB and apoptosis. Additionally may contribute to PEL survival and spindle cell formation	[53,135,136,137,138]
miRNA	Micro RNAs	Contributes to B cell expansion and transformation of rat mesenchymal precursor cells. Downregulates TGFβ signaling and MAF transcription factor. Contributes to cell proliferation and angiogenesis	[139,140,141,142,143]
K15	Viral membrane protein	Induce cell proliferation and angiogenesis. Activates cellular signaling pathways to induces various pro-survival and paracrine-mediated pro-angiogenic cellular cytokines and chemokines, including IL6, IL8, CXCL3, and Cox2	[144,145,146,147,148]
Kaposin B	Kaposin	Regulates cell signaling and reprogramming of vascular endothelial cells	[149,150,151,152]
K1	Variable ITAM-Containing Protein (VIP)	Activates cellular signaling pathways and induces angiogenesis	[60,113,146,153,154,155]
K5	Modulator of immune recognition (MIR2)	Viral E3 ligases capable of ubiquitinating MHC-I, ICAM-1, B7-2, Tetherin (CD317/BST2)	[156,157,158,159]
vIL6	Viral Interleukin-6	Homologues of cellular IL-6. Activate JAK/STAT, MAPK, and PI3K/Akt signaling to induce VEGF pathways to regulate B-cell proliferation	[55,160,161,162,163]
vGPCR	Viral G-protein-coupled receptor (vGPCR)	Homologue of cellular IL-8 receptor. vGPCR activates cellular signaling and induce secretion of proinflammatory cytokines and angiogenic growth factors contributing to angioproliferative tumors	[107,112,164,165,166,167]
vIRF3	Viral interferon regulatory factor-3	Homologues of cellular interferon: Inhibitor of IFN1, p53, NFκB RelA, and p300. Activates HIF-1α and VEGF	[73,168,169,170,171]
vCCL	Viral CC-Chemokine Ligands (vCCLs)	Homologues of cellular chemokines: viral CC-chemokine ligand 1 (vCCL1, (vMIP1), vCCL2 (vMIP2), and vCCL3 (vMIP3), respectively. Modulates signaling through chemokine receptors to promote cell proliferation and angiogenesis	[56,57,172,173]
Glycoprotein B	Glycoprotein	Activates VEGF secretion	[17,74]
K8.1	Glycoprotein	Activates VEGF secretion	[65,74]

**Table 2:** List of commercially available anti-angiogenic drugs for the treatment of KS.

Name of the Drug	Manufacturer	Target	Efficacy	Reference
Imatinib	(Gleevec, Novartis, Basel, Switzerland)	An inhibitor of tyrosine kinases such as Abl, PDGFR and c-kit.	Treatment with imatinib resulted in partial regression of KS tumors in about one-third AIDS-KS patients on combination retroviral therapy	[278]
Sorafenib	(Nexavar, Bayer Healthcare Pharmaceuticals, West Haven, CT, USA)	A small molecule inhibitor of tyrosine-kinases that inhibits VEGFR, PDGFR, FGFR, c-kit, Raf and stem cell factor receptor.	The use of Sorafenib prevented brain metastasis progression and led to unexpected complete remission in a patient with cardiovascular risk and classical KS	[279]
Bevacizumab	(Avastin, Genentech, San Francisco, CA, USA)	Anti-VEGF-A monoclonal antibody that binds to VEGF and neutralizes its action.	The drug induced complete and partial remission of HIV-KS lesions in 3/16 and 2/16 patients respectively, while receiving highly active antiretroviral therapy (HAART)	[280]
Sirolimus	(Rapamune, Pfizer Inc., New York, NY, USA)	Mammalian target of rapamycin (mTOR) inhibitor.	Sirolimus inhibited the progression of dermal KS lesions in kidney-transplant patients being treated with calcineurin inhibitors	[281]

## References

[1] Sturzl M., Zietz C., Monini P., Ensoli B. (2001). Human herpesvirus-8 and kaposi’s sarcoma: Relationship with the multistep concept of tumorigenesis. Adv. Cancer Res..

[2] Verma S.C., Robertson E.S. (2003). Molecular biology and pathogenesis of kaposi sarcoma-associated herpesvirus. FEMS Microbiol. Lett..

[3] Chang Y., Cesarman E., Pessin M.S., Lee F., Culpepper J., Knowles D.M., Moore P.S. (1994). Identification of herpesvirus-like DNA sequences in aids-associated kaposi’s sarcoma. Science.

[4] Cesarman E., Chang Y., Moore P.S., Said J.W., Knowles D.M. (1995). Kaposi’s sarcoma-associated herpesvirus-like DNA sequences in aids-related body-cavity-based lymphomas. N. Engl. J. Med..

[5] Soulier J., Grollet L., Oksenhendler E., Cacoub P., Cazals-Hatem D., Babinet P., d’Agay M.F., Clauvel J.P., Raphael M., Degos L. (1995). Kaposi’s sarcoma-associated herpesvirus-like DNA sequences in multicentric castleman’s disease. Blood.

[6] Deloose S.T., Smit L.A., Pals F.T., Kersten M.J., van Noesel C.J., Pals S.T. (2005). High incidence of kaposi sarcoma-associated herpesvirus infection in HIV-related solid immunoblastic/plasmablastic diffuse large B-cell lymphoma. Leukemia.

[7] Ray A., Marshall V., Uldrick T., Leighty R., Labo N., Wyvill K., Aleman K., Polizzotto M.N., Little R.F., Yarchoan R. (2012). Sequence analysis of kaposi sarcoma-associated herpesvirus (KSHV) microRNAs in patients with multicentric castleman disease and KSHV-associated inflammatory cytokine syndrome. J. Infect. Dis..

[8] Spear P.G., Longnecker R. (2003). Herpesvirus entry: An update. J. Virol..

[9] Lagunoff M., Bechtel J., Venetsanakos E., Roy A.M., Abbey N., Herndier B., McMahon M., Ganem D. (2002). De novo infection and serial transmission of kaposi’s sarcoma-associated herpesvirus in cultured endothelial cells. J. Virol..

[10] Bechtel J.T., Liang Y., Hvidding J., Ganem D. (2003). Host range of kaposi’s sarcoma-associated herpesvirus in cultured cells. J. Virol..

[11] Uppal T., Banerjee S., Sun Z., Verma S.C., Robertson E.S. (2014). KSHV LANA—The master regulator of KSHV latency. Viruses.

[12] DiMaio T.A., Gutierrez K.D., Lagunoff M. (2011). Latent KSHV infection of endothelial cells induces integrin beta3 to activate angiogenic phenotypes. PLoS Pathog..

[13] Bais C., Santomasso B., Coso O., Arvanitakis L., Raaka E.G., Gutkind J.S., Asch A.S., Cesarman E., Gershengorn M.C., Mesri E.A. (1998). G-protein-coupled receptor of kaposi’s sarcoma-associated herpesvirus is a viral oncogene and angiogenesis activator. Nature.

[14] Cannon M., Philpott N.J., Cesarman E. (2003). The kaposi’s sarcoma-associated herpesvirus g protein-coupled receptor has broad signaling effects in primary effusion lymphoma cells. J. Virol..

[15] Cavallin L.E., Goldschmidt-Clermont P., Mesri E.A. (2014). Molecular and cellular mechanisms of KSHV oncogenesis of kaposi’s sarcoma associated with HIV/aids. PLoS Pathog..

[16] DiMaio T.A., Gutierrez K.D., Lagunoff M. (2014). Kaposi’s sarcoma-associated herpesvirus downregulates transforming growth factor beta2 to promote enhanced stability of capillary-like tube formation. J. Virol..

[17] Dai L., Bratoeva M., Toole B.P., Qin Z., Parsons C. (2012). KSHV activation of VEGF secretion and invasion for endothelial cells is mediated through viral upregulation of emmprin-induced signal transduction. Int. J. Cancer.

[18] Qian L.W., Greene W., Ye F., Gao S.J. (2008). Kaposi’s sarcoma-associated herpesvirus disrupts adherens junctions and increases endothelial permeability by inducing degradation of ve-cadherin. J. Virol..

[19] Gbabe O.F., Okwundu C.I., Dedicoat M., Freeman E.E. (2014). Treatment of severe or progressive kaposi’s sarcoma in HIV-infected adults. Cochrane Database Syst. Rev..

[20] Cornali E., Zietz C., Benelli R., Weninger W., Masiello L., Breier G., Tschachler E., Albini A., Sturzl M. (1996). Vascular endothelial growth factor regulates angiogenesis and vascular permeability in kaposi’s sarcoma. Am. J. Pathol..

[21] Gessain A., Duprez R. (2005). Spindle cells and their role in kaposi’s sarcoma. Int. J. Biochem. Cell Biol..

[22] Davis M.A., Sturzl M.A., Blasig C., Schreier A., Guo H.G., Reitz M., Opalenik S.R., Browning P.J. (1997). Expression of human herpesvirus 8-encoded cyclin d in kaposi’s sarcoma spindle cells. J. Natl. Cancer Inst..

[23] Gasperini P., Espigol-Frigole G., McCormick P.J., Salvucci O., Maric D., Uldrick T.S., Polizzotto M.N., Yarchoan R., Tosato G. (2012). Kaposi sarcoma herpesvirus promotes endothelial-to-mesenchymal transition through notch-dependent signaling. Cancer Res..

[24] Ganem D. (2006). KSHV infection and the pathogenesis of kaposi’s sarcoma. Annu Rev. Pathol..

[25] Wang H.W., Trotter M.W., Lagos D., Bourboulia D., Henderson S., Makinen T., Elliman S., Flanagan A.M., Alitalo K., Boshoff C. (2004). Kaposi sarcoma herpesvirus-induced cellular reprogramming contributes to the lymphatic endothelial gene expression in kaposi sarcoma. Nat. Genet..

[26] Hong Y.K., Foreman K., Shin J.W., Hirakawa S., Curry C.L., Sage D.R., Libermann T., Dezube B.J., Fingeroth J.D., Detmar M. (2004). Lymphatic reprogramming of blood vascular endothelium by kaposi sarcoma-associated herpesvirus. Nat. Genet..

[27] Ciufo D.M., Cannon J.S., Poole L.J., Wu F.Y., Murray P., Ambinder R.F., Hayward G.S. (2001). Spindle cell conversion by kaposi’s sarcoma-associated herpesvirus: Formation of colonies and plaques with mixed lytic and latent gene expression in infected primary dermal microvascular endothelial cell cultures. J. Virol..

[28] Moses A.V., Jarvis M.A., Raggo C., Bell Y.C., Ruhl R., Luukkonen B.G., Griffith D.J., Wait C.L., Druker B.J., Heinrich M.C. (2002). Kaposi’s sarcoma-associated herpesvirus-induced upregulation of the c-kit proto-oncogene, as identified by gene expression profiling, is essential for the transformation of endothelial cells. J. Virol..

[29] Grundhoff A., Ganem D. (2004). Inefficient establishment of KSHV latency suggests an additional role for continued lytic replication in kaposi sarcoma pathogenesis. J. Clin. Investig..

[30] Douglas J.L., Gustin J.K., Moses A.V., Dezube B.J., Pantanowitz L. (2010). Kaposi sarcoma pathogenesis: A triad of viral infection, oncogenesis and chronic inflammation. Transl. Biomed..

[31] Dourmishev L.A., Dourmishev A.L., Palmeri D., Schwartz R.A., Lukac D.M. (2003). Molecular genetics of kaposi’s sarcoma-associated herpesvirus (human herpesvirus-8) epidemiology and pathogenesis. Microbiol. Mol. Biol. Rev..

[32] Pantanowitz L., Dezube B.J. (2008). Kaposi sarcoma in unusual locations. BMC Cancer.

[33] Mohanna S., Maco V., Bravo F., Gotuzzo E. (2005). Epidemiology and clinical characteristics of classic kaposi’s sarcoma, seroprevalence, and variants of human herpesvirus 8 in south america: A critical review of an old disease. Int. J. Infect. Dis..

[34] Bhutani M., Polizzotto M.N., Uldrick T.S., Yarchoan R. (2015). Kaposi sarcoma-associated herpesvirus-associated malignancies: Epidemiology, pathogenesis, and advances in treatment. Semin. Oncol..

[35] Horenstein M.G., Moontasri N.J., Cesarman E. (2008). The pathobiology of kaposi’s sarcoma: Advances since the onset of the aids epidemic. J. Cutan Pathol..

[36] Ablashi D.V., Chatlynne L.G., Whitman J.E., Cesarman E. (2002). Spectrum of kaposi’s sarcoma-associated herpesvirus, or human herpesvirus 8, diseases. Clin. Microbiol. Rev..

[37] Nador R.G., Cesarman E., Chadburn A., Dawson D.B., Ansari M.Q., Sald J., Knowles D.M. (1996). Primary effusion lymphoma: A distinct clinicopathologic entity associated with the kaposi’s sarcoma-associated herpes virus. Blood.

[38] Ueda K., Ito E., Karayama M., Ohsaki E., Nakano K., Watanabe S. (2010). KSHV-infected pel cell lines exhibit a distinct gene expression profile. Biochem. Biophys. Res. Commun..

[39] Ueda K., Ohsaki E., Nakano K., Zheng X. (2011). Characterization of kaposi’s sarcoma-associated herpesvirus-related lymphomas by DNA microarray analysis. Leuk. Res. Treat..

[40] Dittmer D.P., Damania B. (2013). Kaposi sarcoma associated herpesvirus pathogenesis (KSHV)—An update. Curr. Opin. Virol..

[41] Webster-Cyriaque J., Duus K., Cooper C., Duncan M. (2006). Oral ebv and KSHV infection in HIV. Adv. Dent. Res..

[42] Sugimoto T., Ito J., Takeda N., Gasyu I., Okazaki T., Sakaguchi M., Osawa N., Tanaka Y., Oka K., Uzu T. (2008). A case of castleman’s disease complicated with nephrotic syndrome due to glomerulopathy mimicking membranoproliferative glomerulonephritis. Am. J. Med. Sci..

[43] Carbone A., Cesarman E., Spina M., Gloghini A., Schulz T.F. (2009). HIV-associated lymphomas and gamma-herpesviruses. Blood.

[44] Bower M., Newsom-Davis T., Naresh K., Merchant S., Lee B., Gazzard B., Stebbing J., Nelson M. (2011). Clinical features and outcome in HIV-associated multicentric castleman’s disease. J. Clin. Oncol..

[45] Carbone A., De Paoli P., Gloghini A., Vaccher E. (2015). KSHV-associated multicentric castleman disease: A tangle of different entities requiring multitarget treatment strategies. Int. J. Cancer.

[46] O’Byrne K.J., Dalqleish A.G., Browning M.J., Steward W.P., Harris A.L. (2000). The relationship between angiogenesis and the immune response in carcinogenesis and the progression of malignant disease. Eur. J. Cancer.

[47] Sakakibara S., Tosato G. (2009). Regulation of angiogenesis in malignancies associated with epstein-barr virus and kaposi’s sarcoma-associated herpes virus. Future Microbiol..

[48] Tandle A., Blazer D.G., Libutti S.K. (2004). Antiangiogenic gene therapy of cancer: Recent developments. J. Transl. Med..

[49] Schulz T.F., Cesarman E. (2015). Kaposi sarcoma-associated herpesvirus: Mechanisms of oncogenesis. Curr. Opin. Virol..

[50] Orenstein J.M. (2008). Ultrastructure of kaposi sarcoma. Ultrastruct. Pathol..

[51] Gramolelli S., Schulz T.F. (2015). The role of kaposi sarcoma-associated herpesvirus in the pathogenesis of kaposi sarcoma. J. Pathol..

[52] Qian L.W., Xie J., Ye F., Gao S.J. (2007). Kaposi’s sarcoma-associated herpesvirus infection promotes invasion of primary human umbilical vein endothelial cells by inducing matrix metalloproteinases. J. Virol..

[53] He M., Zhang W., Bakken T., Schutten M., Toth Z., Jung J.U., Gill P., Cannon M., Gao S.J. (2012). Cancer angiogenesis induced by kaposi sarcoma-associated herpesvirus is mediated by EZH2. Cancer Res..

[54] Bhatt A.P., Damania B. (2012). Aktivation of PI3K/AKT/mTOR signaling pathway by KSHV. Front. Immunol..

[55] Aoki Y., Jaffe E.S., Chang Y., Jones K., Teruya-Feldstein J., Moore P.S., Tosato G. (1999). Angiogenesis and hematopoiesis induced by kaposi’s sarcoma-associated herpesvirus-encoded interleukin-6. Blood.

[56] Boshoff C., Endo Y., Collins P.D., Takeuchi Y., Reeves J.D., Schweickart V.L., Siani M.A., Sasaki T., Williams T.J., Gray P.W. (1997). Angiogenic and HIV-inhibitory functions of KSHV-encoded chemokines. Science.

[57] Stine J.T., Wood C., Hill M., Epp A., Raport C.J., Schweickart V.L., Endo Y., Sasaki T., Simmons G., Boshoff C. (2000). KSHV-encoded CC chemokine VMIP-III is a CCR4 agonist, stimulates angiogenesis, and selectively chemoattracts TH2 cells. Blood.

[58] Liu C., Okruzhnov Y., Li H., Nicholas J. (2001). Human herpesvirus 8 (HHV-8)-encoded cytokines induce expression of and autocrine signaling by vascular endothelial growth factor (VEGF) in HHV-8-infected primary-effusion lymphoma cell lines and mediate VEGF-independent antiapoptotic effects. J. Virol..

[59] Bais C., Van Geelen A., Eroles P., Mutlu A., Chiozzini C., Dias S., Silverstein R.L., Rafii S., Mesri E.A. (2003). Kaposi’s sarcoma associated herpesvirus g protein-coupled receptor immortalizes human endothelial cells by activation of the VEGF receptor-2/ KDR. Cancer Cell.

[60] Wang L., Wakisaka N., Tomlinson C.C., DeWire S.M., Krall S., Pagano J.S., Damania B. (2004). The kaposi’s sarcoma-associated herpesvirus (KSHV/HHV-8) K1 protein induces expression of angiogenic and invasion factors. Cancer Res..

[61] Breen E.C. (2007). VEGF in biological control. J. Cell. Biochem..

[62] Squadrito M.L., De Palma M. (2011). Macrophage regulation of tumor angiogenesis: Implications for cancer therapy. Mol. Aspects Med..

[63] Samaniego F., Markham P.D., Gendelman R., Watanabe Y., Kao V., Kowalski K., Sonnabend J.A., Pintus A., Gallo R.C., Ensoli B. (1998). Vascular endothelial growth factor and basic fibroblast growth factor present in kaposi’s sarcoma (KS) are induced by inflammatory cytokines and synergize to promote vascular permeability and KS lesion development. Am. J. Pathol..

[64] Akula S.M., Ford P.W., Whitman A.G., Hamden K.E., Bryan B.A., Cook P.P., McCubrey J.A. (2005). B-raf-dependent expression of vascular endothelial growth factor-a in kaposi sarcoma-associated herpesvirus-infected human B cells. Blood.

[65] Subramanian R., Sehgal I., D’Auvergne O., Kousoulas K.G. (2010). Kaposi’s sarcoma-associated herpesvirus glycoproteins B and k8.1 regulate virion egress and synthesis of vascular endothelial growth factor and viral interleukin-6 in BCBL-1 cells. J. Virol..

[66] Masood R., Cesarman E., Smith D.L., Gill P.S., Flore O. (2002). Human herpesvirus-8-transformed endothelial cells have functionally activated vascular endothelial growth factor/vascular endothelial growth factor receptor. Am. J. Pathol..

[67] Sivakumar R., Sharma-Walia N., Raghu H., Veettil M.V., Sadagopan S., Bottero V., Varga L., Levine R., Chandran B. (2008). Kaposi’s sarcoma-associated herpesvirus induces sustained levels of vascular endothelial growth factors a and c early during in vitro infection of human microvascular dermal endothelial cells: Biological implications. J. Virol..

[68] Wang L., Damania B. (2008). Kaposi’s sarcoma-associated herpesvirus confers a survival advantage to endothelial cells. Cancer Res..

[69] Lin C., McGough R., Aswad B., Block J.A., Terek R. (2004). Hypoxia induces HIF-1alpha and VEGF expression in chondrosarcoma cells and chondrocytes. J. Orthop. Res..

[70] Brocato J., Chervona Y., Costa M. (2014). Molecular responses to hypoxia-inducible factor 1alpha and beyond. Mol. Pharmacol..

[71] Lang K.J., Kappel A., Goodall G.J. (2002). Hypoxia-inducible factor-1alpha mRNA contains an internal ribosome entry site that allows efficient translation during normoxia and hypoxia. Mol. Biol. Cell.

[72] Carroll P.A., Kenerson H.L., Yeung R.S., Lagunoff M. (2006). Latent kaposi’s sarcoma-associated herpesvirus infection of endothelial cells activates hypoxia-induced factors. J. Virol..

[73] Shin Y.C., Joo C.H., Gack M.U., Lee H.R., Jung J.U. (2008). Kaposi’s sarcoma-associated herpesvirus viral ifn regulatory factor 3 stabilizes hypoxia-inducible factor-1 alpha to induce vascular endothelial growth factor expression. Cancer Res..

[74] Qin Z., Dai L., Slomiany M.G., Toole B.P., Parsons C. (2010). Direct activation of emmprin and associated pathogenesis by an oncogenic herpesvirus. Cancer Res..

[75] Jiang B.H., Liu L.Z. (2009). PI3K/PTEN signaling in angiogenesis and tumorigenesis. Adv. Cancer Res..

[76] Badescu A., Couvelard A., Handra-Luca A. (2014). AKT pathway protein expression in gastrointestinal kaposi sarcomas: Relevance for tumor biology. APMIS.

[77] Anderson K.E., Lipp P., Bootman M., Ridley S.H., Coadwell J., Ronnstrand L., Lennartsson J., Holmes A.B., Painter G.F., Thuring J. (2000). Dapp1 undergoes a PI 3-kinase-dependent cycle of plasma-membrane recruitment and endocytosis upon cell stimulation. Curr. Biol..

[78] Toker A. (2000). Protein kinases as mediators of phosphoinositide 3-kinase signaling. Mol. Pharmacol..

[79] Gingras A.C., Kennedy S.G., O’Leary M.A., Sonenberg N., Hay N. (1998). 4E-BP1, a repressor of mRNA translation, is phosphorylated and inactivated by the AKT(PKB) signaling pathway. Genes Dev..

[80] Beevers C.S., Li F., Liu L., Huang S. (2006). Curcumin inhibits the mammalian target of rapamycin-mediated signaling pathways in cancer cells. Int. J. Cancer.

[81] Hay N., Sonenberg N. (2004). Upstream and downstream of mTOR. Genes Dev..

[82] Hahn-Windgassen A., Nogueira V., Chen C.C., Skeen J.E., Sonenberg N., Hay N. (2005). AKT activates the mammalian target of rapamycin by regulating cellular ATP level and ampk activity. J. Biol. Chem..

[83] Montaner S., Sodhi A., Pece S., Mesri E.A., Gutkind J.S. (2001). The kaposi’s sarcoma-associated herpesvirus G protein-coupled receptor promotes endothelial cell survival through the activation of AKT/protein kinase B. Cancer Res..

[84] Uddin S., Hussain A.R., Al-Hussein K.A., Manogaran P.S., Wickrema A., Gutierrez M.I., Bhatia K.G. (2005). Inhibition of phosphatidylinositol 3’-kinase/AKT signaling promotes apoptosis of primary effusion lymphoma cells. Clin. Cancer Res..

[85] Sin S.H., Roy D., Wang L., Staudt M.R., Fakhari F.D., Patel D.D., Henry D., Harrington W.J., Damania B.A., Dittmer D.P. (2007). Rapamycin is efficacious against primary effusion lymphoma (PEL) cell lines in vivo by inhibiting autocrine signaling. Blood.

[86] Curry C.L., Reed L.L., Golde T.E., Miele L., Nickoloff B.J., Foreman K.E. (2005). Gamma secretase inhibitor blocks notch activation and induces apoptosis in kaposi’s sarcoma tumor cells. Oncogene.

[87] Cheng F., Pekkonen P., Laurinavicius S., Sugiyama N., Henderson S., Gunther T., Rantanen V., Kaivanto E., Aavikko M., Sarek G. (2011). KSHV-initiated notch activation leads to membrane-type-1 matrix metalloproteinase-dependent lymphatic endothelial-to-mesenchymal transition. Cell Host Microbe.

[88] Fischer A., Schumacher N., Maier M., Sendtner M., Gessler M. (2004). The notch target genes Hey1 and Hey2 are required for embryonic vascular development. Genes Dev..

[89] Fischer A., Steidl C., Wagner T.U., Lang E., Jakob P.M., Friedl P., Knobeloch K.P., Gessler M. (2007). Combined loss of Hey1 and Heyl causes congenital heart defects because of impaired epithelial to mesenchymal transition. Circ. Res..

[90] Sharff K.A., Song W.X., Luo X., Tang N., Luo J., Chen J., Bi Y., He B.C., Huang J., Li X. (2009). Hey1 basic helix-loop-helix protein plays an important role in mediating BMP9-induced osteogenic differentiation of mesenchymal progenitor cells. J. Biol. Chem..

[91] Lin G.L., Hankenson K.D. (2011). Integration of bmp, wnt, and notch signaling pathways in osteoblast differentiation. J. Cell. Biochem..

[92] Wang X., He Z., Xia T., Li X., Liang D., Lin X., Wen H., Lan K. (2014). Latency-associated nuclear antigen of kaposi sarcoma-associated herpesvirus promotes angiogenesis through targeting notch signaling effector Hey1. Cancer Res..

[93] Liu G., Yu F.X., Kim Y.C., Meng Z., Naipauer J., Looney D.J., Liu X., Gutkind J.S., Mesri E.A., Guan K.L. (2015). Kaposi sarcoma-associated herpesvirus promotes tumorigenesis by modulating the hippo pathway. Oncogene.

[94] Badouel C., McNeill H. (2011). Snapshot: The hippo signaling pathway. Cell.

[95] Deryugina E.I., Quiqley J.P. (2006). Matrix metalloproteinases and tumor metastasis. Cancer Metastasis Rev..

[96] Impola U., Cuccuru M.A., Masala M.V., Jeskanen L., Cottoni F. (2003). Preliminary communication: Matrix metalloproteinases in kaposi’s sarcoma. Br. J. Dermatol..

[97] Meade-Tollin L.C., Way D., Witte M.H. (1999). Expression of multiple matrix metalloproteinases and urokinase type plasminogen activator in cultured kaposi sarcoma cells. Acta Histochem..

[98] Cianfrocca M., Cooley T.P., Lee J.Y., Rudek M.A., Scadden D.T., Ratner L., Pluda J.M., Figg W.D., Krown S.E., Dezube B.J. (2002). Matrix metalloproteinase inhibitor COL-3 in the treatment of AIDS-related kaposi’s sarcoma: A phase I aids malignancy consortium study. J. Clin. Oncol..

[99] Bergers G., Brekken R., McMahon G., Vu T.H., Itoh T., Tamaki K., Tanzawa K., Thorpe P., Itohara S., Werb Z. (2000). Matrix metalloproteinase-9 triggers the angiogenic switch during carcinogenesis. Nat. Cell Biol..

[100] Ye F.C., Blackbourn D.J., Mengel M., Xie J.P., Qian L.W., Greene W., Yeh I.T., Graham D., Gao S.J. (2007). Kaposi’s sarcoma-associated herpesvirus promotes angiogenesis by inducing angiopoietin-2 expression via AP-1 and ETS1. J. Virol..

[101] Metheny-Barlow L.J., Li L.Y. (2003). The enigmatic role of angiopoietin-1 in tumor angiogenesis. Cell Res..

[102] Fukuhara S., Sako K., Noda K., Zhang J., Minami M., Mochizuki N. (2010). Angiopoietin-1/Tie2 receptor signaling in vascular quiescence and angiogenesis. Histol. Histopathol..

[103] Brown L.F., Dezube B.J., Tognazzi K., Dvorak H.F., Yancopoulos G.D. (2000). Expression of Tie1, Tie2, and angiopoietins 1, 2, and 4 in kaposi’s sarcoma and cutaneous angiosarcoma. Am. J. Pathol..

[104] Zheng X., Ohsaki E., Ueda K. (2015). Mechanism of angiopoietin-1 upregulation in kaposi’s sarcoma-associated herpesvirus-infected PEL cell lines. J. Virol..

[105] Takeda N., Maemura K., Imai Y., Harada T., Kawanami D. (2004). Endothelial PAS domain protein 1 gene promotes angiogenesis through the transactivation of both vascular endothelial growth factor and its receptor. Circ. Res..

[106] Jham B.C., Ma T., Hu J., Chaisuparat R., Friedman E.R., Pandolfi P.P., Schneider A., Sodhi A., Montaner S. (2011). Amplification of the angiogenic signal through the activation of the TSC/mTOR/HIF axis by the KSHV VGPCR in kaposi’s sarcoma. PLoS ONE.

[107] Sodhi A., Chaisuparat R., Hu J., Ramsdell A.K., Manning B.D., Sausville E.A., Sawai E.T., Molinolo A., Gutkind J.S., Montaner S. (2006). The TSC2/mTOR pathway drives endothelial cell transformation induced by the kaposi’s sarcoma-associated herpesvirus G protein-coupled receptor. Cancer Cell.

[108] Ma T., Jham B.C., Hu J., Friedman E.R., Basile J.R., Molinolo A., Sodhi A., Montaner S. (2010). Viral g protein-coupled receptor up-regulates angiopoietin-like 4 promoting angiogenesis and vascular permeability in kaposi’s sarcoma. Proc. Natl. Acad. Sci. USA.

[109] Ma T., Patel H., Babapoor-Farrokhran S., Franklin R., Semenza G.L., Sodhi A., Montaner S. (2015). KSHV induces aerobic glycolysis and angiogenesis through HIF-1-dependent upregulation of pyruvate kinase 2 in kaposi’s sarcoma. Angiogenesis.

[110] Haas D.A., Bala K., Busche G., Weidner-Glunde M., Santag S., Kati S., Gramolelli S., Damas M., Dittrich-Breiholz O., Kracht M. (2013). The inflammatory kinase MAP4K4 promotes reactivation of kaposi’s sarcoma herpesvirus and enhances the invasiveness of infected endothelial cells. PLoS Pathog..

[111] Sharma-Walia N., Paul A.G., Bottero V., Sadagopan S., Veettil M.V., Kerur N., Chandran B. (2010). Kaposi’s sarcoma associated herpes virus (KSHV) induced cox-2: A key factor in latency, inflammation, angiogenesis, cell survival and invasion. PLoS Pathog..

[112] Montaner S., Sodhi A., Molinolo A., Bugge T.H., Sawai E.T., He Y., Li Y., Ray P.E., Gutkind J.S. (2003). Endothelial infection with KSHV genes in vivo reveals that VGPCR initiates kaposi’s sarcomagenesis and can promote the tumorigenic potential of viral latent genes. Cancer Cell.

[113] Tomlinson C.C., Damania B. (2004). The K1 protein of kaposi’s sarcoma-associated herpesvirus activates the AKT signaling pathway. J. Virol..

[114] Morris V.A., Punjabi A.S., Lagunoff M. (2008). Activation of AKT through gp130 receptor signaling is required for kaposi’s sarcoma-associated herpesvirus-induced lymphatic reprogramming of endothelial cells. J. Virol..

[115] Wang C., Zhu C., Wei F., Zhang L., Mo X., Feng Y., Xu J., Yuan Z., Robertson E., Cai Q. (2015). Constitutive activation of interleukin-13/stat6 contributes to kaposi’s sarcoma-associated herpesvirus-related primary effusion lymphoma cell proliferation and survival. J. Virol..

[116] Zhang J., He S., Wang Y., Brulois K., Lan K., Jung J.U., Feng P. (2015). Herpesviral g protein-coupled receptors activate nfat to induce tumor formation via inhibiting the serca calcium atpase. PLoS Pathog..

[117] Di Bartolo D.L., Cannon M., Liu Y.F., Renne R., Chadburn A., Boshoff C., Cesarman E. (2008). KSHV LANA inhibits TGF-beta signaling through epigenetic silencing of the TGF-beta type II receptor. Blood.

[118] Botto S., Totonchy J.E., Gustin J.K., Moses A.V. (2015). Kaposi sarcoma herpesvirus induces HO-1 during de novo infection of endothelial cells via viral miRNA-dependent and -independent mechanisms. MBio.

[119] Sodhi A., Montaner S., Patel V., Zohar M., Bais C., Mesri E.A., Gutkind J.S. (2000). The kaposi’s sarcoma-associated herpes virus G protein-coupled receptor up-regulates vascular endothelial growth factor expression and secretion through mitogen-activated protein kinase and p38 pathways acting on hypoxia-inducible factor 1alpha. Cancer Res..

[120] Angius F., Uda S., Piras E., Spolitu S., Ingianni A., Batetta B., Pompei R. (2015). Neutral lipid alterations in human herpesvirus 8-infected huvec cells and their possible involvement in neo-angiogenesis. BMC Microbiol..

[121] Sun F., Xiao Y., Qu Z. (2015). Oncovirus kaposi sarcoma herpesvirus (KSHV) represses tumor suppressor PDLIM2 to persistently activate nuclear factor kappab (NF-kappaB) and stat3 transcription factors for tumorigenesis and tumor maintenance. J. Biol. Chem..

[122] Dwyer J., Azzi S., Leclair H.M., Georges S., Carlotti A., Treps L., Galan-Moya E.M., Alexia C., Dupin N., Bidere N. (2015). The guanine exchange factor SWAP70 mediates vGPCR-induced endothelial plasticity. Cell Commun. Signal..

[123] Ye F., Lei X., Gao S.J. (2011). Mechanisms of kaposi’s sarcoma-associated herpesvirus latency and reactivation. Adv. Virol..

[124] Gantt S., Casper C. (2011). Human herpesvirus 8-associated neoplasms: The roles of viral replication and antiviral treatment. Curr. Opin. Infect. Dis..

[125] Santag S., Jager W., Karsten C.B., Kati S., Pietrek M., Steinemann D., Sarek G., Ojala P.M., Schulz T.F. (2013). Recruitment of the tumour suppressor protein p73 by kaposi’s sarcoma herpesvirus latent nuclear antigen contributes to the survival of primary effusion lymphoma cells. Oncogene.

[126] Friborg J., Kong W., Hottiger M.O., Nabel G.J. (1999). P53 inhibition by the LANA protein of KSHV protects against cell death. Nature.

[127] Watanabe T., Sugaya M., Atkins A.M., Aquilino E.A., Yang A., Borris D.L., Brady J., Blauvelt A. (2003). Kaposi’s sarcoma-associated herpesvirus latency-associated nuclear antigen prolongs the life span of primary human umbilical vein endothelial cells. J. Virol..

[128] Bubman D., Guasparri I., Cesarman E. (2007). Deregulation of c-Myc in primary effusion lymphoma by kaposi’s sarcoma herpesvirus latency-associated nuclear antigen. Oncogene.

[129] Fujimuro M., Wu F.Y., ApRhys C., Kajumbula H., Young D.B., Hayward G.S., Hayward S.D. (2003). A novel viral mechanism for dysregulation of beta-catenin in kaposi’s sarcoma-associated herpesvirus latency. Nat. Med..

[130] Chang Y., Moore P.S., Talbot S.J., Boshoff C.H., Zarkowska T., Godden K., Paterson H., Weiss R.A., Mittnacht S. (1996). Cyclin encoded by KS herpesvirus. Nature.

[131] Godden-Kent D., Talbot S.J., Boshoff C., Chang Y., Moore P., Weiss R.A., Mittnacht S. (1997). The cyclin encoded by kaposi’s sarcoma-associated herpesvirus stimulates CDK6 to phosphorylate the retinoblastoma protein and histone H1. J. Virol..

[132] Swanton C., Mann D.J., Fleckenstein B., Neipel F., Peters G., Jones N. (1997). Herpes viral cyclin/Cdk6 complexes evade inhibition by CDK inhibitor proteins. Nature.

[133] Verschuren E.W., Hodgson J.G., Gray J.W., Kogan S., Jones N., Evan G.I. (2004). The role of p53 in suppression of KSHV cyclin-induced lymphomagenesis. Cancer Res..

[134] Sarek G., Jarviluoma A., Moore H.M., Tojkander S., Vartia S., Biberfeld P., Laiho M., Ojala P.M. (2010). Nucleophosmin phosphorylation by v-cyclin-CDK6 controls KSHV latency. PLoS Pathog..

[135] Grossmann C., Podgrabinska S., Skobe M., Ganem D. (2006). Activation of NF-kappaB by the latent vFLIP gene of kaposi’s sarcoma-associated herpesvirus is required for the spindle shape of virus-infected endothelial cells and contributes to their proinflammatory phenotype. J. Virol..

[136] Ballon G., Akar G., Cesarman E. (2015). Systemic expression of kaposi sarcoma herpesvirus (KSHV) vFLIP in endothelial cells leads to a profound proinflammatory phenotype and myeloid lineage remodeling in vivo. PLoS Pathog..

[137] Guasparri I., Keller S.A., Cesarman E. (2004). KSHV vFLIP is essential for the survival of infected lymphoma cells. J. Exp. Med..

[138] An J., Sun Y., Sun R., Rettig M.B. (2003). Kaposi’s sarcoma-associated herpesvirus encoded vFLIP induces cellular IL-6 expression: The role of the NF-kappaB and JNK/AP1 pathways. Oncogene.

[139] Umbach J.L., Cullen B.R. (2010). In-depth analysis of kaposi’s sarcoma-associated herpesvirus microRNA expression provides insights into the mammalian microRNA-processing machinery. J. Virol..

[140] Hu M., Wang C., Li W., Lu W., Bai Z., Qin D., Yan Q., Zhu J., Krueger B.J., Renne R. (2015). A KSHV microRNA directly targets g protein-coupled receptor kinase 2 to promote the migration and invasion of endothelial cells by inducing CXCR2 and activating AKT signaling. PLoS Pathog..

[141] Liu Y., Sun R., Lin X., Liang D., Deng Q., Lan K. (2012). Kaposi’s sarcoma-associated herpesvirus-encoded microRNA miR-K12–11 attenuates transforming growth factor beta signaling through suppression of SMAD5. J. Virol..

[142] Samols M.A., Skalsky R.L., Maldonado A.M., Riva A., Lopez M.C., Baker H.V., Renne R. (2007). Identification of cellular genes targeted by KSHV-encoded microRNAs. PLoS Pathog..

[143] Hansen A., Henderson S., Lagos D., Nikitenko L., Coulter E., Roberts S., Gratrix F., Plaisance K., Renne R., Bower M. (2010). KSHV-encoded miRNAs target MAF to induce endothelial cell reprogramming. Genes Dev..

[144] Bala K., Bosco R., Gramolelli S., Haas D.A., Kati S., Pietrek M., Havemeier A., Yakushko Y., Singh V.V., Dittrich-Breiholz O. (2012). Kaposi’s sarcoma herpesvirus K15 protein contributes to virus-induced angiogenesis by recruiting PLCgamma1 and activating NFAT1-dependent RCAN1 expression. PLoS Pathog..

[145] Gramolelli S., Weidner-Glunde M., Abere B., Viejo-Borbolla A., Bala K., Ruckert J., Kremmer E., Schulz T.F. (2015). Inhibiting the recruitment of PLCgamma1 to kaposi’s sarcoma herpesvirus K15 protein reduces the invasiveness and angiogenesis of infected endothelial cells. PLoS Pathog..

[146] Steinbruck L., Gustems M., Medele S., Schulz T.F., Lutter D., Hammerschmidt W. (2015). K1 and K15 of kaposi’s sarcoma-associated herpesvirus are partial functional homologues of latent membrane protein 2A of epstein-barr virus. J. Virol..

[147] Brinkmann M.M., Pietrek M., Dittrich-Breiholz O., Kracht M., Schulz T.F. (2007). Modulation of host gene expression by the K15 protein of kaposi’s sarcoma-associated herpesvirus. J. Virol..

[148] Havemeier A., Gramolelli S., Pietrek M., Jochmann R., Sturzl M., Schulz T.F. (2014). Activation of NF-kappaB by the kaposi’s sarcoma-associated herpesvirus K15 protein involves recruitment of the NF-kappaB-inducing kinase, IkappaB kinases, and phosphorylation of p65. J. Virol..

[149] Corcoran J.A., Johnston B.P., McCormick C. (2015). Viral activation of MK2-hsp27-p115RhoGEF-RhoA signaling axis causes cytoskeletal rearrangements, p-body disruption and are-mRNA stabilization. PLoS Pathog..

[150] King C.A. (2013). Kaposi’s sarcoma-associated herpesvirus kaposin B induces unique monophosphorylation of stat3 at serine 727 and MK2-mediated inactivation of the stat3 transcriptional repressor trim28. J. Virol..

[151] McCormick C., Ganem D. (2005). The kaposin b protein of KSHV activates the p38/MK2 pathway and stabilizes cytokine mRNAs. Science.

[152] Yoo J., Kang J., Lee H.N., Aguilar B., Kafka D., Lee S., Choi I., Lee J., Ramu S., Haas J. (2010). Kaposin-b enhances the prox1 mRNA stability during lymphatic reprogramming of vascular endothelial cells by kaposi’s sarcoma herpes virus. PLoS Pathog..

[153] Wang L., Dittmer D.P., Tomlinson C.C., Fakhari F.D., Damania B. (2006). Immortalization of primary endothelial cells by the K1 protein of kaposi’s sarcoma-associated herpesvirus. Cancer Res..

[154] Wang S., Wang S., Maeng H., Young D.P., Prakash O., Fayad L.E., Younes A., Samaniego F. (2007). K1 protein of human herpesvirus 8 suppresses lymphoma cell Fas-mediated apoptosis. Blood.

[155] Lee H., Veazey R., Williams K., Li M., Guo J., Neipel F., Fleckenstein B., Lackner A., Desrosiers R.C., Jung J.U. (1998). Deregulation of cell growth by the K1 gene of kaposi’s sarcoma-associated herpesvirus. Nat. Med..

[156] Ishido S., Wang C., Lee B.S., Cohen G.B., Jung J.U. (2000). Downregulation of major histocompatibility complex class i molecules by kaposi’s sarcoma-associated herpesvirus K3 and K5 proteins. J. Virol..

[157] Coscoy L., Ganem D. (2001). A viral protein that selectively downregulates ICAM-1 and B7-2 and modulates T cell costimulation. J. Clin. Investig..

[158] Ishido S., Choi J.K., Lee B.S., Wang C., DeMaria M., Johnson R.P., Cohen G.B., Jung J.U. (2000). Inhibition of natural killer cell-mediated cytotoxicity by kaposi’s sarcoma-associated herpesvirus K5 protein. Immunity.

[159] Mansouri M., Viswanathan K., Douglas J.L., Hines J., Gustin J., Moses A.V., Fruh K. (2009). Molecular mechanism of BST2/tetherin downregulation by K5/MIR2 of kaposi’s sarcoma-associated herpesvirus. J. Virol..

[160] Moore P.S., Boshoff C., Weiss R.A., Chang Y. (1996). Molecular mimicry of human cytokine and cytokine response pathway genes by KSHV. Science.

[161] Molden J., Chang Y., You Y., Moore P.S., Goldsmith M.A. (1997). A kaposi’s sarcoma-associated herpesvirus-encoded cytokine homolog (vIL-6) activates signaling through the shared gp130 receptor subunit. J. Biol. Chem..

[162] Burger R., Neipel F., Fleckenstein B., Savino R., Ciliberto G., Kalden J.R., Gramatzki M. (1998). Human herpesvirus type 8 interleukin-6 homologue is functionally active on human myeloma cells. Blood.

[163] Cousins E., Gao Y., Sandford G., Nicholas J. (2014). Human herpesvirus 8 viral interleukin-6 signaling through gp130 promotes virus replication in primary effusion lymphoma and endothelial cells. J. Virol..

[164] Mutlu A.D., Cavallin L.E., Vincent L., Chiozzini C., Eroles P., Duran E.M., Asgari Z., Hooper A.T., La Perle K.M., Hilsher C. (2007). In vivo-restricted and reversible malignancy induced by human herpesvirus-8 KSHV: A cell and animal model of virally induced kaposi’s sarcoma. Cancer Cell.

[165] Emuss V., Lagos D., Pizzey A., Gratrix F., Henderson S.R., Boshoff C. (2009). KSHV manipulates notch signaling by DLL4 and JAG1 to alter cell cycle genes in lymphatic endothelia. PLoS Pathog..

[166] Sodhi A., Montaner S., Patel V., Gomez-Roman J.J., Li Y., Sausville E.A., Sawai E.T., Gutkind J.S. (2004). AKT plays a central role in sarcomagenesis induced by kaposi’s sarcoma herpesvirus-encoded g protein-coupled receptor. Proc. Natl. Acad. Sci. USA.

[167] Martin D., Galisteo R., Molinolo A.A., Wetzker R., Hirsch E., Gutkind J.S. (2011). PI3Kgamma mediates kaposi’s sarcoma-associated herpesvirus vGPCR-induced sarcomagenesis. Cancer Cell.

[168] Rivas C., Thlick A.E., Parravicini C., Moore P.S., Chang Y. (2001). Kaposi’s sarcoma-associated herpesvirus LANA2 is a B-cell-specific latent viral protein that inhibits p53. J. Virol..

[169] Wies E., Mori Y., Hahn A., Kremmer E., Sturzl M., Fleckenstein B., Neipel F. (2008). The viral interferon-regulatory factor-3 is required for the survival of KSHV-infected primary effusion lymphoma cells. Blood.

[170] Esteban M., Garcia M.A., Domingo-Gil E., Arroyo J., Nombela C., Rivas C. (2003). The latency protein LANA2 from kaposi’s sarcoma-associated herpesvirus inhibits apoptosis induced by dsRNA-activated protein kinase but not RNAse l activation. J. Gen. Virol..

[171] Wies E., Hahn A.S., Schmidt K., Viebahn C., Rohland N., Lux A., Schellhorn T., Holzer A., Jung J.U., Neipel F. (2009). The kaposi’s sarcoma-associated herpesvirus-encoded vIRF-3 inhibits cellular IRF-5. J. Biol. Chem..

[172] Endres M.J., Garlisi C.G., Xiao H., Shan L., Hedrick J.A. (1999). The kaposi’s sarcoma-related herpesvirus (KSHV)-encoded chemokine vMIP-I is a specific agonist for the cc chemokine receptor (CCR)8. J. Exp. Med..

[173] Weber K.S., Grone H.J., Rocken M., Klier C., Gu S., Wank R., Proudfoot A.E., Nelson P.J., Weber C. (2001). Selective recruitment of TH2-type cells and evasion from a cytotoxic immune response mediated by viral macrophage inhibitory protein-II. Eur. J. Immunol..

[174] Chen W., Hilton I.B., Staudt M.R., Burd C.E., Dittmer D.P. (2010). Distinct p53, p53:LANA, and LANA complexes in kaposi’s sarcoma—Associated herpesvirus lymphomas. J. Virol..

[175] Radkov S.A., Kellam P., Boshoff C. (2000). The latent nuclear antigen of kaposi sarcoma-associated herpesvirus targets the retinoblastoma-E2F pathway and with the oncogene hras transforms primary rat cells. Nat. Med..

[176] Liu J., Martin H.J., Liao G., Hayward S.D. (2007). The kaposi’s sarcoma-associated herpesvirus LANA protein stabilizes and activates c-Myc. J. Virol..

[177] Paudel N., Sadagopan S., Balasubramanian S., Chandran B. (2012). Kaposi’s sarcoma-associated herpesvirus latency-associated nuclear antigen and angiogenin interact with common host proteins, including annexin A2, which is essential for survival of latently infected cells. J. Virol..

[178] Sadagopan S., Valiya Veettil M., Paudel N., Bottero V., Chandran B. (2011). Kaposi’s sarcoma-associated herpesvirus-induced angiogenin plays roles in latency via the phospholipase C gamma pathway: Blocking angiogenin inhibits latent gene expression and induces the lytic cycle. J. Virol..

[179] Sharma-Walia N., Patel K., Chandran K., Marginean A., Bottero V., Kerur N., Paul A.G. (2012). Cox-2/pge2: Molecular ambassadors of kaposi’s sarcoma-associated herpes virus oncoprotein-v-FLIP. Oncogenesis.

[180] Sturzl M., Hohenadl C., Zietz C., Castanos-Velez E., Wunderlich A., Ascherl G., Biberfeld P., Monini P., Browning P.J., Ensoli B. (1999). Expression of K13/v-FLIP gene of human herpesvirus 8 and apoptosis in kaposi’s sarcoma spindle cells. J. Natl. Cancer Inst..

[181] Kwun H.J., da Silva S.R., Shah I.M., Blake N., Moore P.S., Chang Y. (2007). Kaposi’s sarcoma-associated herpesvirus latency-associated nuclear antigen 1 mimics epstein-barr virus EBNA1 immune evasion through central repeat domain effects on protein processing. J. Virol..

[182] Zaldumbide A., Ossevoort M., Wiertz E.J., Hoeben R.C. (2007). In cis inhibition of antigen processing by the latency-associated nuclear antigen I of kaposi sarcoma herpes virus. Mol. Immunol..

[183] Li X., Liang D., Lin X., Robertson E.S., Lan K. (2011). Kaposi’s sarcoma-associated herpesvirus-encoded latency-associated nuclear antigen reduces interleukin-8 expression in endothelial cells and impairs neutrophil chemotaxis by degrading nuclear p65. J. Virol..

[184] Cloutier N., Flamand L. (2010). Kaposi sarcoma-associated herpesvirus latency-associated nuclear antigen inhibits interferon (IFN) beta expression by competing with IFN regulatory factor-3 for binding to IFNB promoter. J. Biol. Chem..

[185] Kwun H.J., da Silva S.R., Qin H., Ferris R.L., Tan R., Chang Y., Moore P.S. (2011). The central repeat domain 1 of kaposi’s sarcoma-associated herpesvirus (KSHV) latency associated-nuclear antigen 1 (LANA1) prevents cis MHC class i peptide presentation. Virology.

[186] Thakker S., Purushothaman P., Gupta N., Challa S., Cai Q., Verma S.C. (2015). Kaposi’s sarcoma-associated herpesvirus latency-associated nuclear antigen inhibits major histocompatibility complex class II expression by disrupting enhanceosome assembly through binding with the regulatory factor x complex. J. Virol..

[187] Gregory S.M., Wang L., West J.A., Dittmer D.P., Damania B. (2012). Latent kaposi’s sarcoma-associated herpesvirus infection of monocytes downregulates expression of adaptive immune response costimulatory receptors and proinflammatory cytokines. J. Virol..

[188] Cai Q., Murakami M., Si H., Robertson E.S. (2007). A potential alpha-helix motif in the amino terminus of LANA encoded by kaposi’s sarcoma-associated herpesvirus is critical for nuclear accumulation of HIF-1alpha in normoxia. J. Virol..

[189] Cai Q., Lan K., Verma S.C., Si H., Lin D., Robertson E.S. (2006). Kaposi’s sarcoma-associated herpesvirus latent protein LANA interacts with HIF-1 alpha to upregulate rta expression during hypoxia: Latency control under low oxygen conditions. J. Virol..

[190] Li M., Lee H., Yoon D.W., Albrecht J.C., Fleckenstein B., Neipel F., Jung J.U. (1997). Kaposi’s sarcoma-associated herpesvirus encodes a functional cyclin. J. Virol..

[191] Godfrey A., Anderson J., Papanastasiou A., Takeuchi Y., Boshoff C. (2005). Inhibiting primary effusion lymphoma by lentiviral vectors encoding short hairpin rna. Blood.

[192] Grundhoff A., Ganem D. (2001). Mechanisms governing expression of the v-FLIP gene of kaposi’s sarcoma-associated herpesvirus. J. Virol..

[193] Bieleski L., Talbot S.J. (2001). Kaposi’s sarcoma-associated herpesvirus vcyclin open reading frame contains an internal ribosome entry site. J. Virol..

[194] Ellis M., Chew Y.P., Fallis L., Freddersdorf S., Boshoff C., Weiss R.A., Lu X., Mittnacht S. (1999). Degradation of p27(Kip) cdk inhibitor triggered by kaposi’s sarcoma virus cyclin-cdk6 complex. EMBO J..

[195] Jarviluoma A., Ojala P.M. (2006). Cell signaling pathways engaged by KSHV. Biochim. Biophys. Acta.

[196] Verschuren E.W., Jones N., Evan G.I. (2004). The cell cycle and how it is steered by kaposi’s sarcoma-associated herpesvirus cyclin. J. Gen. Virol..

[197] Bertin J., Armstrong R.C., Ottilie S., Martin D.A., Wang Y., Banks S., Wang G.H., Senkevich T.G., Alnemri E.S., Moss B. (1997). Death effector domain-containing herpesvirus and poxvirus proteins inhibit both Fas- and TNFR1-induced apoptosis. Proc. Natl. Acad. Sci. USA.

[198] Hu S., Vincenz C., Buller M., Dixit V.M. (1997). A novel family of viral death effector domain-containing molecules that inhibit both CD-95- and tumor necrosis factor receptor-1-induced apoptosis. J. Biol. Chem..

[199] Thome M., Schneider P., Hofmann K., Fickenscher H., Meinl E., Neipel F., Mattmann C., Burns K., Bodmer J.L., Schroter M. (1997). Viral FLICE-inhibitory proteins (FLIPs) prevent apoptosis induced by death receptors. Nature.

[200] Chaudhary P.M., Jasmin A., Eby M.T., Hood L. (1999). Modulation of the NF-kappaB pathway by virally encoded death effector domains-containing proteins. Oncogene.

[201] Chugh P., Matta H., Schamus S., Zachariah S., Kumar A., Richardson J.A., Smith A.L., Chaudhary P.M. (2005). Constitutive NF-kappaB activation, normal Fas-induced apoptosis, and increased incidence of lymphoma in human herpes virus 8 K13 transgenic mice. Proc. Natl. Acad. Sci. USA.

[202] Matta H., Chaudhary P.M. (2004). Activation of alternative NF-kappaB pathway by human herpes virus 8-encoded Fas-associated death domain-like IL-1 beta-converting enzyme inhibitory protein (vFLIP). Proc. Natl. Acad. Sci. USA.

[203] Douglas J., Dutia B., Rhind S., Stewart J.P., Talbot S.J. (2004). Expression in a recombinant murid herpesvirus 4 reveals the in vivo transforming potential of the K1 open reading frame of kaposi’s sarcoma-associated herpesvirus. J. Virol..

[204] Pfeffer S., Sewer A., Lagos-Quintana M., Sheridan R., Sander C., Grasser F.A., van Dyk L.F., Ho C.K., Shuman S., Chien M. (2005). Identification of microRNAs of the herpesvirus family. Nat. Methods.

[205] Samols M.A., Hu J., Skalsky R.L., Renne R. (2005). Cloning and identification of a microRNA cluster within the latency-associated region of kaposi’s sarcoma-associated herpesvirus. J. Virol..

[206] Wu X.J., Pu X.M., Zhao Z.F., Zhao Y.N., Kang X.J., Wu W.D., Zou Y.M., Wu C.Y., Qu Y.Y., Zhang D.Z. (2015). The expression profiles of microRNAs in kaposi’s sarcoma. Tumour Biol..

[207] Cai X., Lu S., Zhang Z., Gonzalez C.M., Damania B., Cullen B.R. (2005). Kaposi’s sarcoma-associated herpesvirus expresses an array of viral microRNAs in latently infected cells. Proc. Natl. Acad. Sci. USA.

[208] Catrina Ene A.M., Borze I., Guled M., Costache M., Leen G., Sajin M., Ionica E., Chitu A., Knuutila S. (2014). MicroRNA expression profiles in kaposi’s sarcoma. Pathol. Oncol. Res..

[209] Viollet C., Davis D.A., Reczko M., Ziegelbauer J.M., Pezzella F., Ragoussis J., Yarchoan R. (2015). Next-generation sequencing analysis reveals differential expression profiles of miRNA-mRNA target pairs in KSHV-infected cells. PLoS ONE.

[210] Chugh P.E., Sin S.H., Ozgur S., Henry D.H., Menezes P., Griffith J., Eron J.J., Damania B., Dittmer D.P. (2013). Systemically circulating viral and tumor-derived microRNAs in KSHV-associated malignancies. PLoS Pathog..

[211] Feldman E.R., Kara M., Coleman C.B., Grau K.R., Oko L.M., Krueger B.J., Renne R., van Dyk L.F., Tibbetts S.A. (2014). Virus-encoded microRNAs facilitate gammaherpesvirus latency and pathogenesis in vivo. MBio.

[212] Gallaher A.M., Das S., Xiao Z., Andresson T., Kieffer-Kwon P., Happel C., Ziegelbauer J. (2013). Proteomic screening of human targets of viral microRNAs reveals functions associated with immune evasion and angiogenesis. PLoS Pathog..

[213] Forte E., Raja A.N., Shamulailatpam P., Manzano M., Schipma M.J., Casey J.L., Gottwein E. (2015). MicroRNA-mediated transformation by the kaposi’s sarcoma-associated herpesvirus kaposin locus. J. Virol..

[214] Ramalingam D., Happel C., Ziegelbauer J.M. (2015). Kaposi’s sarcoma-associated herpesvirus microRNAs repress breakpoint cluster region protein expression, enhance RAC1 activity, and increase in vitro angiogenesis. J. Virol..

[215] Quinn S.R., O’Neill L.A. (2014). The role of microRNAs in the control and mechanism of action of IL-10. Curr. Top. Microbiol. Immunol..

[216] Yoo J., Lee H.N., Choi I., Choi D., Chung H.K., Kim K.E., Lee S., Aguilar B., Kang J., Park E. (2012). Opposing regulation of PROX1 by interleukin-3 receptor and notch directs differential host cell fate reprogramming by kaposi sarcoma herpes virus. PLoS Pathog..

[217] Pyakurel P., Pak F., Mwakigonja A.R., Kaaya E., Heiden T., Biberfeld P. (2006). Lymphatic and vascular origin of kaposi’s sarcoma spindle cells during tumor development. Int. J. Cancer.

[218] Choi J.K., Lee B.S., Shim S.N., Li M., Jung J.U. (2000). Identification of the novel K15 gene at the rightmost end of the kaposi’s sarcoma-associated herpesvirus genome. J. Virol..

[219] Sharp T.V., Wang H.W., Koumi A., Hollyman D., Endo Y., Ye H., Du M.Q., Boshoff C. (2002). K15 protein of kaposi’s sarcoma-associated herpesvirus is latently expressed and binds to HAX-1, a protein with antiapoptotic function. J. Virol..

[220] Tsai Y.H., Wu M.F., Wu Y.H., Chang S.J., Lin S.F., Sharp T.V., Wang H.W. (2009). The M type K15 protein of kaposi’s sarcoma-associated herpesvirus regulates microRNA expression via its SH2-binding motif to induce cell migration and invasion. J. Virol..

[221] Brinkmann M.M., Glenn M., Rainbow L., Kieser A., Henke-Gendo C., Schulz T.F. (2003). Activation of mitogen-activated protein kinase and nf-kappab pathways by a kaposi’s sarcoma-associated herpesvirus K15 membrane protein. J. Virol..

[222] Cho N.H., Choi Y.K., Choi J.K. (2008). Multi-transmembrane protein K15 of kaposi’s sarcoma-associated herpesvirus targets Lyn kinase in the membrane raft and induces NFAT/AP1 activities. Exp. Mol. Med..

[223] Choi Y.B., Nicholas J. (2008). Autocrine and paracrine promotion of cell survival and virus replication by human herpesvirus 8 chemokines. J. Virol..

[224] Wang L., Brinkmann M.M., Pietrek M., Ottinger M., Dittrich-Breiholz O., Kracht M., Schulz T.F. (2007). Functional characterization of the M-type K15-encoded membrane protein of kaposi’s sarcoma-associated herpesvirus. J. Gen. Virol..

[225] Lee B.S., Lee S.H., Feng P., Chang H., Cho N.H., Jung J.U. (2005). Characterization of the kaposi’s sarcoma-associated herpesvirus K1 signalosome. J. Virol..

[226] Prakash O., Swamy O.R., Peng X., Tang Z.Y., Li L., Larson J.E., Cohen J.C., Gill J., Farr G., Wang S. (2005). Activation of src kinase Lyn by the kaposi sarcoma-associated herpesvirus K1 protein: Implications for lymphomagenesis. Blood.

[227] Wang J.F., Liu Z.Y., Anand A.R., Zhang X., Brown L.F., Dezube B.J., Gill P., Ganju R.K. (2004). Alpha-chemokine-mediated signal transduction in human kaposi’s sarcoma spindle cells. Biochim. Biophys. Acta.

[228] Prakash O., Tang Z.Y., Peng X., Coleman R., Gill J., Farr G., Samaniego F. (2002). Tumorigenesis and aberrant signaling in transgenic mice expressing the human herpesvirus-8 K1 gene. J. Natl. Cancer Inst..

[229] Lee B.S., Alvarez X., Ishido S., Lackner A.A., Jung J.U. (2000). Inhibition of intracellular transport of B cell antigen receptor complexes by kaposi’s sarcoma-associated herpesvirus K1. J. Exp. Med..

[230] Yao S., Hu M., Hao T., Li W., Xue X., Xue M., Zhu X., Zhou F., Qin D., Yan Q. (2015). MiRNA-891a-5p mediates HIV-1 tat and KSHV ORF-K1 synergistic induction of angiogenesis by activating NF-kappaB signaling. Nucleic Acids Res..

[231] Lagunoff M., Lukac D.M., Ganem D. (2001). Immunoreceptor tyrosine-based activation motif-dependent signaling by kaposi’s sarcoma-associated herpesvirus K1 protein: Effects on lytic viral replication. J. Virol..

[232] Lee H., Guo J., Li M., Choi J.K., DeMaria M., Rosenzweig M., Jung J.U. (1998). Identification of an immunoreceptor tyrosine-based activation motif of K1 transforming protein of kaposi’s sarcoma-associated herpesvirus. Mol. Cell Biol..

[233] Rezza G., Andreoni M., Dorrucci M., Pezzotti P., Monini P., Zerboni R., Salassa B., Colangeli V., Sarmati L., Nicastri E. (1999). Human herpesvirus 8 seropositivity and risk of kaposi’s sarcoma and other acquired immunodeficiency syndrome-related diseases. J. Natl. Cancer Inst..

[234] Aoki Y., Tosato G. (2003). Pathogenesis and manifestations of human herpesvirus-8-associated disorders. Semin. Hematol..

[235] Xue M., Yao S., Hu M., Li W., Hao T., Zhou F., Zhu X., Lu H., Qin D., Yan Q. (2014). HIV-1 NEF and KSHV oncogene K1 synergistically promote angiogenesis by inducing cellular miR-718 to regulate the PTEN/AKT/mTOR signaling pathway. Nucleic Acids Res..

[236] Coscoy L., Sanchez D.J., Ganem D. (2001). A novel class of herpesvirus-encoded membrane-bound E3 ubiquitin ligases regulates endocytosis of proteins involved in immune recognition. J. Cell Biol..

[237] Coscoy L., Ganem D. (2000). Kaposi’s sarcoma-associated herpesvirus encodes two proteins that block cell surface display of MHC class I chains by enhancing their endocytosis. Proc. Natl. Acad. Sci. USA.

[238] Brulois K., Jung J.U. (2014). Interplay between kaposi’s sarcoma-associated herpesvirus and the innate immune system. Cytokine Growth Factor Rev..

[239] Manes T.D., Hoer S., Muller W.A., Lehner P.J., Pober J.S. (2010). Kaposi’s sarcoma-associated herpesvirus K3 and K5 proteins block distinct steps in transendothelial migration of effector memory CD4+ T cells by targeting different endothelial proteins. J. Immunol..

[240] Mansouri M., Douglas J., Rose P.P., Gouveia K., Thomas G., Means R.E., Moses A.V., Fruh K. (2006). Kaposi sarcoma herpesvirus K5 removes CD31/pecam from endothelial cells. Blood.

[241] Mansouri M., Rose P.P., Moses A.V., Fruh K. (2008). Remodeling of endothelial adherens junctions by kaposi’s sarcoma-associated herpesvirus. J. Virol..

[242] Li Q., Means R., Lang S., Jung J.U. (2007). Downregulation of gamma interferon receptor 1 by kaposi’s sarcoma-associated herpesvirus K3 and K5. J. Virol..

[243] Karki R., Lang S.M., Means R.E. (2011). The march family E3 ubiquitin ligase K5 alters monocyte metabolism and proliferation through receptor tyrosine kinase modulation. PLoS Pathog..

[244] Neipel F., Albrecht J.C., Ensser A., Huang Y.Q., Li J.J., Friedman-Kien A.E., Fleckenstein B. (1997). Human herpesvirus 8 encodes a homolog of interleukin-6. J. Virol..

[245] Nicholas J., Ruvolo V.R., Burns W.H., Sandford G., Wan X., Ciufo D., Hendrickson S.B., Guo H.G., Hayward G.S., Reitz M.S. (1997). Kaposi’s sarcoma-associated human herpesvirus-8 encodes homologues of macrophage inflammatory protein-1 and interleukin-6. Nat. Med..

[246] Zhu X., Guo Y., Yao S., Yan Q., Xue M., Hao T., Zhou F., Zhu J., Qin D., Lu C. (2014). Synergy between kaposi’s sarcoma-associated herpesvirus (KSHV) VIL-6 and HIV-1 nef protein in promotion of angiogenesis and oncogenesis: Role of the AKT signaling pathway. Oncogene.

[247] Hideshima T., Chauhan D., Teoh G., Raje N., Treon S.P., Tai Y.T., Shima Y., Anderson K.C. (2000). Characterization of signaling cascades triggered by human interleukin-6 versus kaposi’s sarcoma-associated herpes virus-encoded viral interleukin 6. Clin. Cancer Res..

[248] Osborne J., Moore P.S., Chang Y. (1999). KSHV-encoded viral IL-6 activates multiple human IL-6 signaling pathways. Hum. Immunol..

[249] Sun R., Lin S.F., Staskus K., Gradoville L., Grogan E., Haase A., Miller G. (1999). Kinetics of kaposi’s sarcoma-associated herpesvirus gene expression. J. Virol..

[250] Mesri E.A., Cesarman E., Boshoff C. (2010). Kaposi’s sarcoma and its associated herpesvirus. Nat. Rev. Cancer.

[251] Aoki Y., Tosato G. (1999). Role of vascular endothelial growth factor/vascular permeability factor in the pathogenesis of kaposi’s sarcoma-associated herpesvirus-infected primary effusion lymphomas. Blood.

[252] Ensoli B., Sturzl M. (1998). Kaposi’s sarcoma: A result of the interplay among inflammatory cytokines, angiogenic factors and viral agents. Cytokine Growth Factor Rev..

[253] Guo H.G., Browning P., Nicholas J., Hayward G.S., Tschachler E., Jiang Y.W., Sadowska M., Raffeld M., Colombini S., Gallo R.C. (1997). Characterization of a chemokine receptor-related gene in human herpesvirus 8 and its expression in kaposi’s sarcoma. Virology.

[254] Arvanitakis L., Geras-Raaka E., Varma A., Gershengorn M.C., Cesarman E. (1997). Human herpesvirus KSHV encodes a constitutively active G-protein-coupled receptor linked to cell proliferation. Nature.

[255] Sodhi A., Montaner S., Gutkind J.S. (2004). Does dysregulated expression of a deregulated viral GPCR trigger kaposi’s sarcomagenesis?. FASEB J..

[256] Cesarman E., Nador R.G., Bai F., Bohenzky R.A., Russo J.J., Moore P.S., Chang Y., Knowles D.M. (1996). Kaposi’s sarcoma-associated herpesvirus contains G protein-coupled receptor and cyclin D homologs which are expressed in kaposi’s sarcoma and malignant lymphoma. J. Virol..

[257] Cannon M. (2007). The KSHV and other human herpesviral G protein-coupled receptors. Curr. Top. Microbiol. Immunol..

[258] De Munnik S.M., Smit M.J., Leurs R., Vischer H.F. (2015). Modulation of cellular signaling by herpesvirus-encoded G protein-coupled receptors. Front. Pharmacol..

[259] Shan B., Morris C.A., Zhuo Y., Shelby B.D., Levy D.R., Lasky J.A. (2007). Activation of prommp-2 and SRC by HHV8 vGPCR in human pulmonary arterial endothelial cells. J. Mol. Cell. Cardiol..

[260] Cunningham C., Barnard S., Blackbourn D.J., Davison A.J. (2003). Transcription mapping of human herpesvirus 8 genes encoding viral interferon regulatory factors. J. Gen. Virol..

[261] Jenner R.G., Alba M.M., Boshoff C., Kellam P. (2001). Kaposi’s sarcoma-associated herpesvirus latent and lytic gene expression as revealed by DNA arrays. J. Virol..

[262] Munoz-Fontela C., Collado M., Rodriguez E., Garcia M.A., Alvarez-Barrientos A., Arroyo J., Nombela C., Rivas C. (2005). Identification of a nuclear export signal in the KSHV latent protein LANA2 mediating its export from the nucleus. Exp. Cell Res..

[263] Lubyova B., Kellum M.J., Frisancho J.A., Pitha P.M. (2007). Stimulation of c-Myc transcriptional activity by vIRF-3 of kaposi sarcoma-associated herpesvirus. J. Biol. Chem..

[264] Baresova P., Musilova J., Pitha P.M., Lubyova B. (2014). P53 tumor suppressor protein stability and transcriptional activity are targeted by kaposi’s sarcoma-associated herpesvirus-encoded viral interferon regulatory factor 3. Mol. Cell Biol..

[265] Marcos-Villar L., Lopitz-Otsoa F., Gallego P., Munoz-Fontela C., Gonzalez-Santamaria J., Campagna M., Shou-Jiang G., Rodriguez M.S., Rivas C. (2009). Kaposi’s sarcoma-associated herpesvirus protein LANA2 disrupts pml oncogenic domains and inhibits PML-mediated transcriptional repression of the survivin gene. J. Virol..

[266] Haque N.S., Fallon J.T., Taubman M.B., Harpel P.C. (2001). The chemokine receptor CCR8 mediates human endothelial cell chemotaxis induced by I-309 and kaposi sarcoma herpesvirus-encoded vMIP-I and by lipoprotein(a)-stimulated endothelial cell conditioned medium. Blood.

[267] Pekkonen P., Jarviluoma A., Zinovkina N., Cvrljevic A., Prakash S., Westermarck J., Evan G.I., Cesarman E., Verschuren E.W., Ojala P.M. (2014). KSHV viral cyclin interferes with T-cell development and induces lymphoma through CDK6 and notch activation in vivo. Cell Cycle.

[268] Zhi H., Zahoor M.A., Shudofsky A.M., Giam C.Z. (2015). KSHV vcyclin counters the senescence/G1 arrest response triggered by NF-kappaB hyperactivation. Oncogene.

[269] Eklund L., Bry M., Alitalo K. (2013). Mouse models for studying angiogenesis and lymphangiogenesis in cancer. Mol. Oncol..

[270] Wang L.X., Kang G., Kumar P., Lu W., Li Y., Zhou Y., Li Q., Wood C. (2014). Humanized-blt mouse model of kaposi’s sarcoma-associated herpesvirus infection. Proc. Natl. Acad. Sci. USA.

[271] Zhang J., Zhu L., Lu X., Feldman E.R., Keyes L.R., Wang Y., Fan H., Feng H., Xia Z., Sun J. (2015). Recombinant murine gamma herpesvirus 68 carrying KSHV G protein-coupled receptor induces angiogenic lesions in mice. PLoS Pathog..

[272] Berkova Z., Wang S., Sehgal L., Patel K.P., Prakash O., Samaniego F. (2015). Lymphoid hyperplasia and lymphoma in KSHV K1 transgenic mice. Histol. Histopathol..

[273] Ojala P.M., Schulz T.F. (2014). Manipulation of endothelial cells by KSHV: Implications for angiogenesis and aberrant vascular differentiation. Semin. Cancer Biol..

[274] Ashlock B.M., Ma Q., Issac B., Mesri E.A. (2014). Productively infected murine kaposi’s sarcoma-like tumors define new animal models for studying and targeting KSHV oncogenesis and replication. PLoS ONE.

[275] Carmeliet P., Jain R.K. (2011). Molecular mechanisms and clinical applications of angiogenesis. Nature.

[276] De Falco S. (2014). Antiangiogenesis therapy: An update after the first decade. Korean J. Intern. Med..

[277] Kanno T., Uehara T., Osawa M., Fukumoto H., Mine S., Ueda K., Hasegawa H., Katano H. (2015). Fumagillin, a potent angiogenesis inhibitor, induces kaposi sarcoma-associated herpesvirus replication in primary effusion lymphoma cells. Biochem. Biophys. Res. Commun..

[278] Yeh J.R., Mohan R., Crews C.M. (2000). The antiangiogenic agent TNP-470 requires p53 and p21CIP/WAF for endothelial cell growth arrest. Proc. Natl. Acad. Sci. USA.

[279] Ardavanis A., Doufexis D., Kountourakis P., Rigatos G. (2008). A kaposi’s sarcoma complete clinical response after sorafenib administration. Ann. Oncol..

[280] Uldrick T.S., Wyvill K.M., Kumar P., O’Mahony D., Bernstein W., Aleman K., Polizzotto M.N., Steinberg S.M., Pittaluga S., Marshall V. (2012). Phase ii study of bevacizumab in patients with hiv-associated kaposi’s sarcoma receiving antiretroviral therapt. J. Clin Oncol..

[281] Krown S.E., Roy D., Lee J.Y., Dezube B.J., Reid E.G., Venkataramanan R., Han K., Cesarman E., Dittmer D.P. (2012). Rapamycin with antiretroviral therapy in aids-associated kaposi sarcoma: An aids malignancy consortium study. J. Acquir. Immune Defic. Syndr..

